# Supplementary material for: Wavelength-multiplexed multi-mode EUV reflection ptychography based on automatic differentiation
Source: Light Sci Appl. 2024 Aug 19;13:196. doi: 10.1038/s41377-024-01558-3 (PMC11333750; doi:10.1038/s41377-024-01558-3)
Supplement: Supplementary file 1 — Supplementary Information [file 41377_2024_1558_MOESM1_ESM.pdf]

Supplementary Information for Wavelength-multiplexed Multi-mode EUV Reflection Ptychography based on Automatic-Differentiation

Yifeng Shao[1,2*†], Sven Weerdenburg[1†], Jacob Seifert[2†], H. Paul Urbach[1], Allard P. Mosk[2] and Wim Coene[1,3]

[1]Imaging Physics Department, Applied Science Faculty, Delft University of Technology, Lorentzweg 1, Delft, 2628 CJ , The Netherlands.
[2]Nanophotonics, Debye Institute for Nanomaterials Science and Center for Extreme Matter and Emergent Phenomena, Utrecht University, P.O. Box 80000, Utrecht, 3508 TA, The Netherlands.
[3]Research Department, ASML Netherlands B.V, De Run 6501, Veldhoven, 5504 DR , The Netherlands.

*Corresponding author(s). E-mail(s): y.shao@tudelft.nl;
Contributing authors: S.Weerdenburg@tudelft.nl; j.seifert@uu.nl;
†These authors contributed equally to this work.

Springer Nature 2021 LaTeX template

Contents

1 Wavelength-multiplexed Multi-mode Diffractive Imaging 3

2 Gradient computation with chain rule 4

3 The propagation sub-model 4
3.1 Filtered angular spectrum (AS) propagation 5
3.2 Fresnel (FR) Propagation 6
3.3 Fraunhofer (FH) Propagation 6

4 The chirp z-transform 6

5 Speed comparison on Fast Fourier transform 9

6 Correcting diffraction pattern for propagation between tilted planes 10

7 Background subtraction and reconstruction 14

8 Optimiser and optimisation strategy 15

9 Regularisation and regularisation strategy 17
9.1 Regularisation using L1-norm 17
9.2 Regularisation using Total Variation (TV) 18
9.3 Probe Support Constraint Regularization 19

10 Calculation of the deviation angle between harmonics 20

11 Refocusing 22

12 Orthogonal probe spatial modes 23

13 Spatial frequency analysis for gratings 26

14 Processing of reference AFM measurement 26

15 Rigorous sample multilayer simulation 27

16 Relative height retrieval from reconstructed object phase 28

17 Error analysis against mixed noise and systematic error 31

18 Determining the height of wafer structures 35

1 Wavelength-multiplexed Multi-mode Diffractive Imaging

At the microscopic level, the probe and the object can be interpreted as ensembles of randomly fluctuating functions of positions, whose macroscopic coherence properties can be characterised by the cross-spectral density (CSD), representing the correlation between these functions at a pair of positions for a particular wavelength.

The CSD of the probe can be written as

$$J_P(\lambda;\ \mathbf{r}_1, \mathbf{r}_2) = \langle P(\lambda;\ \mathbf{r}_1) P(\lambda;\ \mathbf{r}_2)^* \rangle \tag{1}$$

where $*$ denotes the complex-conjugate, $\langle\ \rangle$ denotes the ensemble average, λ is the wavelength, and $\mathbf{r}_1$ and $\mathbf{r}_2$ are a pair of positions in the sample plane. As proposed by Wolf in [1], the CSD $J_P(\lambda;\ \mathbf{r}_1, \mathbf{r}_2)$ can be rewritten in the form of a weighted sum as

$$J_P(\lambda;\ \mathbf{r}_1, \mathbf{r}_2) = \sum_m w_{p,m} p_m(\lambda;\ \mathbf{r}_1) p_m(\lambda;\ \mathbf{r}_2)^*, \tag{2}$$

where $w_{p,m}$ and $p_m(\lambda;\ \mathbf{r})$ are the eigenvalues and the eigenfunctions, respectively. The CSD $J_P(\lambda;\ \mathbf{r}_1, \mathbf{r}_2)$ is actually a Hilbert-Schmidt kernel. Similarly, by applying eigendecomposition to the CSD of the object, we obtain

$$J_O(\lambda;\ \mathbf{r}_1, \mathbf{r}_2) = \langle O(\lambda;\ \mathbf{r}_1) O(\lambda;\ \mathbf{r}_2)^* \rangle = \sum_n w_{o,n} o_n(\lambda;\ \mathbf{r}_1) o_n(\lambda;\ \mathbf{r}_2)^*, \tag{3}$$

where $w_{o,n}$ denotes the eigenvalue and $o_n(\lambda;\ \mathbf{r})$ denotes the eigenfunction.

Because the CSD of the probe and the object are statistically independent of each other, we can thus derive the CSD of the exit field, which was modelled as $\Phi_k(\mathbf{r}) = \sigma_k P(\mathbf{r}) O(\mathbf{r} - \mathbf{r}_k)$ in the spatially coherent case, as follows:

$$\begin{aligned} J_{\Phi_k}(\lambda, \mathbf{r}_1, \mathbf{r}_2) &= J_{P_k}(\lambda;\ \mathbf{r}_1, \mathbf{r}_2) J_{O_k}(\lambda;\ \mathbf{r}_1, \mathbf{r}_2) \\ &= \sum_m \sum_n \left[\sigma_k P_m(\lambda;\ \mathbf{r}_1) O_n(\lambda;\ \mathbf{r}_1 - \mathbf{r}_k)\right] \left[\sigma_k P_m(\lambda;\ \mathbf{r}_2) O_n(\lambda;\ \mathbf{r}_2 - \mathbf{r}_k)\right]^*, \end{aligned}$$

where $P_m(\lambda;\ \mathbf{r}) = \sqrt{w_{p,m}} p_m(\lambda;\ \mathbf{r})$ and $O_n(\lambda;\ \mathbf{r}) = \sqrt{w_{o,n}} o_n(\lambda;\ \mathbf{r})$ are referred to as the spatial modes of the probe and the object, respectively. σ_k and $\mathbf{r}_k$ denote the relative power in the probe and the scanning position, respectively, for the kth acquisition. We can further derive the CSD of the diffracted field, expressed as

$$J_{\Psi_k}(\boldsymbol{\rho}_1, \boldsymbol{\rho}_2) = \left\langle \mathcal{D}_{\lambda,z} \left[\Phi_k(\mathbf{r}_1)\right](\boldsymbol{\rho}_1) \mathcal{D}_{\lambda,z} \left[\Phi_k(\mathbf{r}_2)\right]^* (\boldsymbol{\rho}_2) \right\rangle, \tag{4}$$

where $\mathcal{D}_{\lambda,z}$ denotes the propagator for propagation distance z and wavelength λ. Finally, by selecting some of the harmonic wavelengths emitted by the HHG

source to contribute to the diffraction pattern through spectral filtering, we can derive the expression of the diffraction pattern for dispersive objects as:

$$\begin{aligned} I_k(\boldsymbol{\rho}) &= \mathcal{M}\left[J_{\Psi_k}(\boldsymbol{\rho},\boldsymbol{\rho}) + \mathcal{N}(\boldsymbol{\rho})\right] \\ &= \mathcal{M}\left[\sum_{l=1}^{L}\sum_{m=1}^{M}\sum_{n=1}^{N}\left|\mathcal{D}_{\lambda_l,z}\left[\sigma_k P_{l,m}(\mathbf{r}) O_{l,n}(\mathbf{r}-\mathbf{s}_k)\right](\boldsymbol{\rho})\right|^2 + \mathcal{N}(\boldsymbol{\rho})\right], \end{aligned} \tag{5}$$

where $\mathcal{M}$ denotes the operator that applies the measurement-related effects to the diffraction pattern, and $\mathcal{N}(\boldsymbol{\rho})$ denotes the background signal intensity on the camera. For non-dispersive objects, the sample modes become independent of the wavelength, and hence the object reduces from $O_{l,n}(\mathbf{r})$ to $O_n(\mathbf{r})$ in Eq. 5.

2 Gradient computation with chain rule

In the update scheme for iterative optimisation, automatic-differentiation (AD) uses the chain rule to compute the gradients of the variables with respect to the loss function that depends on the overall model $\mathbf{f}$. Essentially, AD accumulates the Jacobians (matrices of partial derivatives) of each sub-model in reverse order of evaluating $\mathbf{f}$, following the dashed arrows shown in Fig. 1 (B) in the article.

Note that the mask function $\mathcal{M}$ is considered to be fixed and does not participate in the optimisation process. The explicit formulas for computing all gradients are listed below, according to the computational graph in Fig. 1 (B) in the article:

$$\begin{aligned} \frac{\partial\mathcal{L}}{\partial\mathcal{N}} &= \mathbf{J}_{\mathcal{L}}\mathbf{J}_5^{\mathcal{N}}, & \frac{\partial\mathcal{L}}{\partial\mathbf{O}} &= \mathbf{J}_{\mathcal{L}}\mathbf{J}_5\mathbf{J}_4\mathbf{J}_3\mathbf{J}_2^{\mathbf{O}}, \\ \frac{\partial\mathcal{L}}{\partial\lambda} &= \mathbf{J}_{\mathcal{L}}\mathbf{J}_5\mathbf{J}_4^{\lambda}, & \frac{\partial\mathcal{L}}{\partial\mathbf{s}_k} &= \mathbf{J}_{\mathcal{L}}\mathbf{J}_5\mathbf{J}_4\mathbf{J}_3\mathbf{J}_2, \\ \frac{\partial\mathcal{L}}{\partial z} &= \mathbf{J}_{\mathcal{L}}\mathbf{J}_5\mathbf{J}_4^{z}, & \frac{\partial\mathcal{L}}{\partial\mathbf{P}} &= \mathbf{J}_{\mathcal{L}}\mathbf{J}_5\mathbf{J}_4\mathbf{J}_3\mathbf{J}_1^{\mathbf{P}}, \\ & & \frac{\partial\mathcal{L}}{\partial\sigma_k} &= \mathbf{J}_{\mathcal{L}}\mathbf{J}_5\mathbf{J}_4\mathbf{J}_3\mathbf{J}_1, \end{aligned} \tag{6}$$

where $\mathbf{J}_{\mathcal{L}}$ is the Jacobian of the loss function with respect to the kth acquisition of the diffraction pattern, $\mathbf{J}_i$ is the Jacobian of $\mathbf{f}_i$, where $i = 1, 2, 3, 4, 5$, consisting of the partial derivatives of its output with respect to its input, and $\mathbf{J}_i^{\boldsymbol{\theta}_i}$ denotes the Jacobian for the specific variable $\boldsymbol{\theta}_i$ in $\mathbf{f}_i$.

3 The propagation sub-model

This sub-model computes the field propagation from the sample to the camera in free space, which evaluates the following function:

$$\Psi = \mathbf{f}(\Phi, \Delta\boldsymbol{\rho}, \Delta\mathbf{r}) \tag{7}$$

where Φ and Ψ are 4-dimensional arrays, both with shape (L, MN, Y, X), representing the input exit field and the output diffracted field, respectively. $\Delta\mathbf{r} = (\Delta x, \Delta y)$ and $\Delta\boldsymbol{\rho} = (\Delta u, \Delta v)$ are sampling intervals, with equal numbers of sampling points Y and X.

We write the propagator per spectral harmonic per spatial mode as

$$\Psi(\boldsymbol{\rho}) = \iint \mathcal{D}_{\lambda,\Delta z}(\boldsymbol{\rho}, \mathbf{r})\Phi(\mathbf{r})\mathrm{d}^2\mathbf{r}, \tag{8}$$

where $\mathbf{r}$ and $\boldsymbol{\rho}$ are the coordinates in the sample plane and the camera plane, respectively, and $\mathcal{D}_{\lambda,\Delta z}(\boldsymbol{\rho}, \mathbf{r})$ is the kernel of the propagation. Our algorithm implements three methods for computing the free-space field propagation in the angular spectrum (AS), the Fresnel (FR), and the Fraunhofer (FH) region, respectively. The Fourier transform in each method can be computed by either the fast Fourier transform (FFT) or the chirp z-transform (CZT) [2–5].

3.1 Filtered angular spectrum (AS) propagation

In the AS region, the number sampling points in both planes are equal and we can rewrite the propagator kernel Eq. 8 as [6, 7]

$$\mathcal{D}_{\lambda,\Delta z}(\boldsymbol{\rho}, \mathbf{r}) = \frac{\exp(ikR)}{R}\frac{z}{R}\left[\frac{1}{2\pi R} + \frac{1}{i\lambda}\right], \tag{9}$$

where $R = \sqrt{|\boldsymbol{\rho} - \mathbf{r}|^2 + \Delta z^2}$ is the relative distance between $\boldsymbol{\rho}$ and $\mathbf{r}$, and $k = 2\pi/\lambda$ is the wave number. Eq. 8 can be computed as a convolution since the propagation kernel only depends on $\boldsymbol{\rho} - \mathbf{r}$:

$$\begin{aligned}\Psi(\boldsymbol{\rho}) &= \iint \mathcal{D}_{\lambda,\Delta z}(\boldsymbol{\rho} - \mathbf{r})\Phi(\mathbf{r})\mathrm{d}^2\mathbf{r} \\ &= \mathcal{F}^{-1}\left[\mathcal{F}\left[\mathcal{D}_{\lambda,\Delta z}(\mathbf{r})\right](\mathbf{k})\mathcal{F}\left[\Phi(\mathbf{r})\right](\mathbf{k})\right](\boldsymbol{\rho}),\end{aligned} \tag{10}$$

where $\mathcal{F}$ and $\mathcal{F}^{-1}$ denote the Fourier transform and its inverse, respectively, and $\mathbf{k}$ is the transverse component of the 3-dimensional (3D) wave vector $\mathbf{K}$ that relates to the equally sampled 2-dimensional coordinate vector $\mathbf{r}$ and $\boldsymbol{\rho}$. We can derive the Fourier transformed propagation kernel given by

$$\mathcal{F}\left[\mathcal{D}_{\lambda,\Delta z}(\mathbf{r})\right](\mathbf{k}) = \exp\left[ik_z(\mathbf{k}, \lambda)\Delta z\right], \tag{11}$$

where k_z denotes the z component of the wave vector $\mathbf{K}$ and is defined as

$$k_z(\mathbf{k}, \lambda) = \begin{cases} \sqrt{k_0^2 - |\mathbf{k}|^2} & \text{if } |\mathbf{k}| \leq k_0 = \frac{2\pi}{\lambda} \\ 0 & \text{otherwise} \end{cases} \tag{12}$$

To avoid any aliasing effect, we implement the band-limited AS method [7], which uses zero-padding to solve the issue of periodic boundary conditions and applies a filter to the propagation kernel in the k-space.

3.2 Fresnel (FR) Propagation

In the FR region, we assume that the samplings in both planes are related to each other by the Shannon-Nyquist sampling theorem, and under the paraxial approximation $|\boldsymbol{\rho} - \mathbf{r}| \ll \Delta z$, we can write the propagation kernel as

$$\begin{aligned}\mathcal{D}_{\lambda,\Delta z}(\boldsymbol{\rho},\mathbf{r}) &= \exp\left(\frac{i\pi}{\lambda\Delta z}|\boldsymbol{\rho}-\mathbf{r}|^2\right)\\ &= \exp\left(\frac{i\pi}{\lambda\Delta z}|\boldsymbol{\rho}|^2\right)\exp\left(\frac{i\pi}{\lambda\Delta z}|\mathbf{r}|^2\right)\exp\left(\frac{-i2\pi}{\lambda\Delta z}\boldsymbol{\rho}\cdot\mathbf{r}\right),\end{aligned} \tag{13}$$

and hence Eq. 8 becomes

$$\begin{aligned}\Psi(\boldsymbol{\rho}) &= \exp\left(\frac{i\pi}{\lambda\Delta z}|\boldsymbol{\rho}|^2\right)\iint \Phi(\mathbf{r})\exp\left(\frac{i\pi}{\lambda\Delta z}|\mathbf{r}|^2\right)\exp\left(\frac{-i2\pi}{\lambda\Delta z}\boldsymbol{\rho}\cdot\mathbf{r}\right)\mathrm{d}^2\mathbf{r}\\ &= \exp\left(i\pi\lambda\Delta z|\bar{\boldsymbol{\rho}}|^2\right)\mathcal{F}\left[\Phi(\mathbf{r})\exp\left(\frac{i\pi}{\lambda\Delta z}|\mathbf{r}|^2\right)\right](\bar{\boldsymbol{\rho}}),\end{aligned} \tag{14}$$

where $\bar{\boldsymbol{\rho}} = \boldsymbol{\rho}/(\lambda\Delta z)$ is the normalised coordinate in the camera plane. Due to the Shannon-Nyquist sampling theorem, $\bar{\boldsymbol{\rho}}$ is equivalent to the coordinate of the spatial frequency of $\mathbf{r}$ in the sample plane. We have defined the following relation between the spatial frequency and the wave vector: $2\pi\bar{\boldsymbol{\rho}} = \mathbf{k}$.

3.3 Fraunhofer (FH) Propagation

In the FH region, the samplings in both planes are related to each other by the Shannon-Nyquist sampling theorem, and under the paraxial approximation $|\boldsymbol{\rho} - \mathbf{r}| \ll \Delta z$ and $|\mathbf{r}| \ll \Delta z$, we can write the propagation kernel as

$$\mathcal{D}_{\lambda,\Delta z}(\boldsymbol{\rho},\mathbf{r}) = \exp\left(\frac{i\pi}{\lambda\Delta z}|\boldsymbol{\rho}|^2\right)\exp\left(\frac{-i2\pi}{\lambda\Delta z}\boldsymbol{\rho}\cdot\mathbf{r}\right), \tag{15}$$

and hence Eq. 8 becomes

$$\Psi(\boldsymbol{\rho}) = \exp\left(i\pi\lambda\Delta z|\bar{\boldsymbol{\rho}}|^2\right)\mathcal{F}\left[\Phi(\mathbf{r})\right](\bar{\boldsymbol{\rho}}), \tag{16}$$

where $\bar{\boldsymbol{\rho}} = \boldsymbol{\rho}/(\lambda\Delta z)$, and the relation $2\pi\bar{\boldsymbol{\rho}} = \mathbf{k}$ still holds.

4 The chirp z-transform

The computation of the Fourier transform is at the core of all propagators. Recall the one-dimensional discrete Fourier transform (DFT), which can be expressed as

$$g_q = \sum_p f_p \exp\left(-i2\pi x_p u_q\right), \tag{17}$$

where f_p and g_p denote the discretised functions before and after the DFT, respectively, and the sampling points are given by

$$\begin{aligned} x_p &= x_0 + p\Delta x, \quad p = 0, \cdots, N_x - 1 \\ u_q &= u_0 + q\Delta u, \quad q = 0, \cdots, N_u - 1, \end{aligned} \tag{18}$$

where x_0, u_0 are the starting locations, Δ_x, Δ_u are the sampling intervals, and N_x, N_u are the sampling numbers before and after the DFT, respectively. Substituting Eq. 18 into Eq. 17 yields that

$$\begin{aligned} g_q &= \sum_p f_p \exp\left[-i2\pi(x_0 + p\Delta x)(u_0 + q\Delta u)\right] \\ &= \exp(-i2\pi x_0 q\Delta u) \sum_p f_p \exp(-i2\pi u_0 p\Delta x)\exp(-i2\pi pq\Delta x\Delta u), \end{aligned} \tag{19}$$

in which the constant phase factor $\exp(-i2\pi x_0 u_0)$ has been neglected. In Eq. 19, two exponential terms $\exp(-i2\pi x_0 q\Delta u)$ and $\exp(-i2\pi u_0 p\Delta x)$ can be compensated by shifting f_p and g_q before and after the DFT, respectively.

In the literature, the chirp z transform (CZT) algorithm is proposed as an alternative to the Fast Fourier transform (FFT) algorithm for computing the DFT to relax the stringent sampling requirements while maintaining computation efficiency. By replacing the product pq in Eq. 19 with the identity

$$pq = \frac{q^2}{2} + \frac{p^2}{2} - \frac{(q-p)^2}{2}, \tag{20}$$

we can rewrite the DFT (with the two exponential terms compensated by shifting) as

$$g_q = \sum_p f_p \exp\left[-i2\pi\Delta x\Delta u\left(\frac{q^2}{2} + \frac{p^2}{2} - \frac{(q-p)^2}{2}\right)\right] \tag{21}$$

Notice that Eq. 21 is essentially a convolution that can be written as

$$\begin{aligned} g_q &= A_q \sum_p f_p B_p C_{q-p}, \\ &= A_q \mathcal{F}^{-1}\left[\mathcal{F}[f_p B_p]\mathcal{F}[C_p]\right] \end{aligned} \tag{22}$$

where

$$
\begin{aligned}
A_q &= \exp\left(-i2\pi\Delta x\Delta u\frac{q^2}{2}\right),\\
B_p &= \exp\left(-i2\pi\Delta x\Delta u\frac{p^2}{2}\right),\\
C_p &= \exp\left(+i2\pi\Delta x\Delta u\frac{p^2}{2}\right).
\end{aligned}
\tag{23}
$$

By computing the convolution in Eq. 22 using the FFT algorithm, the efficiency of the CZT algorithm is guaranteed. The efficiency can be further improved by computing and storing the term $\mathcal{F}[C_p]$ in advance. Zero-padding, described in detail in [2, 3], is required to solve the issue of periodic boundary conditions when computing the convolution using the FFT algorithm. Eq. 21 indicates that the sampling interval $\Delta x, \Delta u$ and the sampling number N_x, N_u can both be arbitrary. However, the Shannon-Nyqvist Sampling theorem still needs to be satisfied.

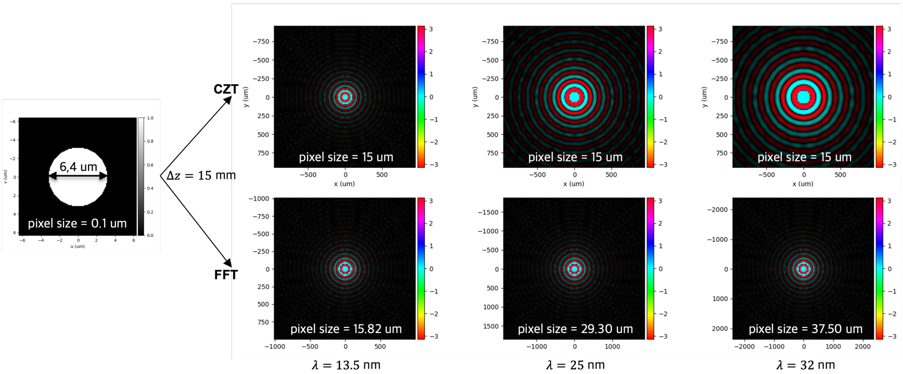

Fig. S1 The far-field diffraction pattern of an aperture computed with the CZT algorithm (top) and with the FFT algorithm (below).

In Fig. S1 we compare the CZT with the FFT by simulating the far-field Fraunhofer diffraction of an aperture with a diameter of 6.4 um for a distance of 15 mm at 3 wavelengths: 13.5 nm, 25 nm, 32 nm. In the sample plane, we used pixel size 0.1 um and pixel number 1024 in both directions. Fig. S1 demonstrates that the CZT algorithm can compute diffraction patterns on sampling grids with the same pixel size for different wavelengths. In practical situations, this is advantageous compared to the case of using the FFT algorithm because only one set of sampling grids is needed in the camera plane for all wavelengths.

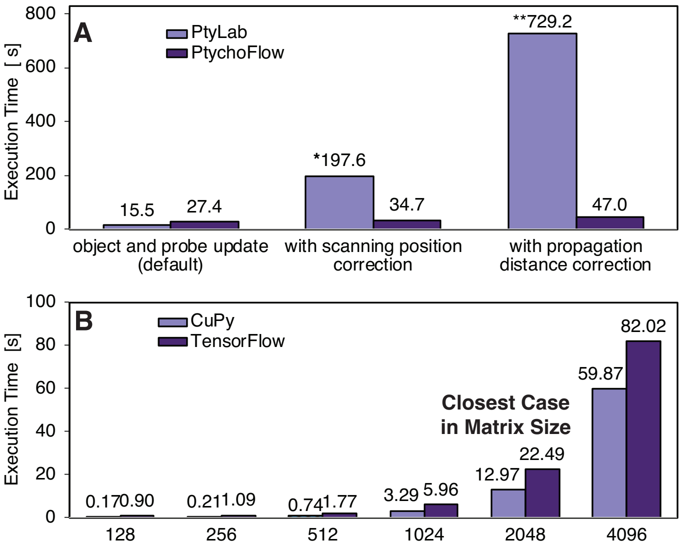

Fig. S2 Comparison of execution time for ptychography reconstruction (A) and FFT (B). Cupy and TensorFlow are the numerical modules that PtyLab and PtychoFlow use to compute the FFT.

5 Speed comparison on Fast Fourier transform

Computing the Fast Fourier transform (FFT) plays an essential role in ptychography as all propagators are interpreted as Fourier transforms and are calculated by the FFT method. Note that the CZT method also uses FFT for the convolution. In this computation, we show that despite both using the CUDA library to accelerate the computation of FFT by GPU, we find a noticeable performance difference between the different implementations in CuPy (PtyLab) and TensorFlow (PtychoFlow).

We benchmark the execution time of FFT computation for a series of square random matrices with various sizes in 64-bit complex precision. For reproducibility, we use a fixed random seed for the comparison. In each case, we repeat the FFT computation 11 times. The first execution time is neglected because it may contain the time required to allocate memory on GPU or build the computational graph. The benchmark is performed on a Nvidia RTX A6000 GPU without any other processes running in parallel.

Fig. S2 (B) shows that compared with TensorFlow, CuPy is optimised better for GPU acceleration and achieves faster performance for FFT computation in all cases. In the case closest to the experimental dataset, CuPy is about 2 times faster than TensorFlow, which is in agreement with the speed comparison for the default object and probe update in Fig. S2 (A). The default update does not involve any correction features, so the update schemes of both algorithms should be identical. As a result, only the speed of FFT computation matters in the speed comparison.

6 Correcting diffraction pattern for propagation between tilted planes

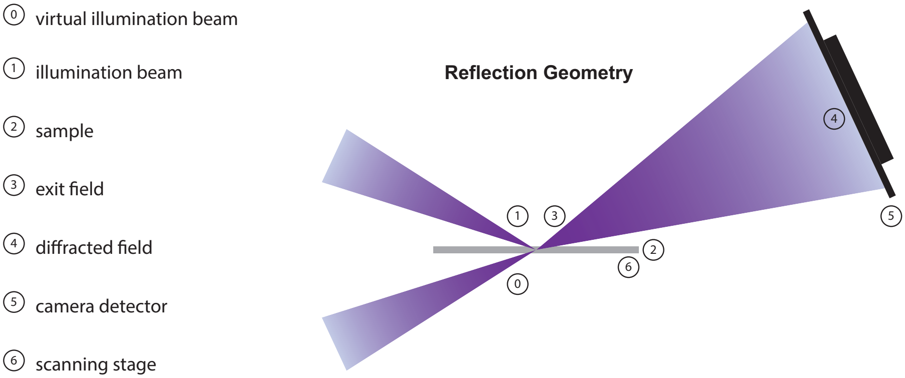

Fig. S3 Diffractive imaging in reflection geometry depicted in transmission geometry with a tilted sample.

Fig. S3 illustrates that diffractive imaging in reflection geometry can be depicted as in transmission geometry by mirroring the illumination beam over the sample, leading to a sample tilt with respect to the illumination beam at an angle equal to the angle of incidence (AOI) between the illumination beam and the surface normal of the sample. Due to the conical diffraction, we cannot describe the FR/FH propagation from the tilted sample to the camera by Fourier transformation. Instead, interpolation is required to relate the equidistantly sampled fields in both planes.

In Fig. S4, we illustrate an example of sample tilt for an illumination beam incident at an angle of ϕ_{inc} in the x-z plane. The intermediate plane is defined as the plane obtained by rotating the sample plane around the y-axis by an angle of $\phi_y = \phi_{inc}$ such that the intermediate plane and the camera plane are parallel to each other. Now let us consider the following transformation matrix $\mathbf{T}$ for arbitrary rotation between the coordinates:

$$\mathbf{R}' = \mathbf{T}\mathbf{R}, \tag{24}$$

$$\mathbf{R} = \mathbf{T}^{-1}\mathbf{R}', \tag{25}$$

where $\mathbf{R} = (x, y, z)$ and $\mathbf{R}' = (x', y', z')$ are the 3D coordinates of locations in the sample plane and the intermediate plane, respectively, with shared origins, and $\mathbf{T}$ denotes the transformation matrix given by [8, 9]

$$\mathbf{T} = \mathbf{T}_x(\phi_x)\mathbf{T}_y(\phi_y)\mathbf{T}_z(\phi_z),$$

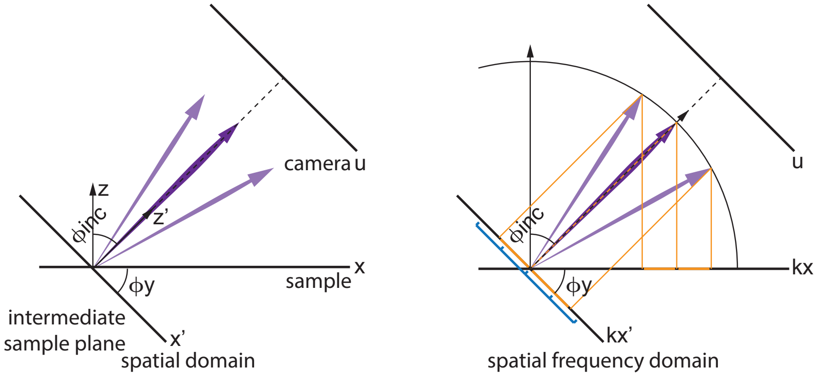

Fig. S4 Illustration of light propagation between tilted planes. The purple arrows represent the spatial frequency components of the sample plane field, which can be sampled in both k_x (orange, uniform) and $k'_x(k_x)$ (orange, non-uniform) coordinates, which are related by coordinate transformation. The diffracted field, which is sampled in the coordinate $u' = u/(\lambda\Delta z)$ (blue, uniform), is equal to the angular spectrum of the field in the sample plane. Distorted diffraction patterns can be corrected by interpolating from u' to $k'_x(k_x)/(2\pi)$. Starting with known coordinates u in the camera plane and k_x in the reciprocal space of the sample plane, we generate u' by scaling and k'_x by rotation transformation. Finally, we interpolate the diffraction pattern from u' to $k'_x(k_x)/(2\pi)$.

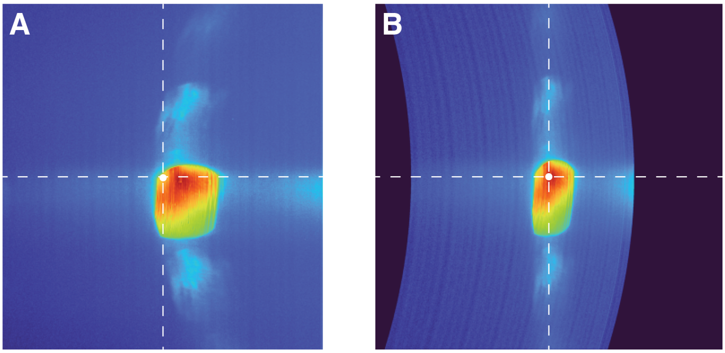

Fig. S5 Illustration of diffraction pattern before (**A**) and after (**B**) correction. The illustrated diffraction pattern is the average of 225 diffraction patterns used in the reconstruction, normalised to between zero and one. The dashed lines indicate the horizontal and vertical axes and the solid dot represents the origin of the camera coordinate. The correction requires the zeroth diffraction order aligned at the origin and it depends on the propagation distance and the tilt angle. In **B**, only pixels inside the FOV contribute to the optimisation, while other pixels outside will be masked.

$$= \begin{bmatrix} T_{11} & T_{12} & T_{13} \\ T_{21} & T_{22} & T_{23} \\ T_{31} & T_{32} & T_{33} \end{bmatrix}, \tag{26}$$

where $\mathbf{T}_\xi(\phi_\xi)$ represents the transformation matrix for the rotation around the ξ-axis by an angle of ϕ_ξ, where $\xi = x, y, z$. The transformation matrix is unitary by definition, i.e.,

$$\mathbf{T}^{-1} = \mathbf{T}^{\mathrm{t}}, \tag{27}$$

where -1 and t denote the inverse and the transpose of $\mathbf{T}$, respectively, and

$$(\mathbf{TA}) \cdot (\mathbf{TB}) = \mathbf{A} \cdot \mathbf{B}, \tag{28}$$

where $\mathbf{A}$ and $\mathbf{B}$ are both arbitrary vectors in the three-dimensional space.

Recall the expression for the FR/FH propagator between parallel planes Eq. 14 and Eq. 16, both of which involve a Fourier transform that can be expressed as

$$\Psi(\bar{\boldsymbol{\rho}}) = \iint \Phi(\mathbf{r}') e^{-i2\pi\bar{\boldsymbol{\rho}}\cdot\mathbf{r}'} \mathrm{d}^2\mathbf{r}', \tag{29}$$

where we have neglected the quadratic phase factor in the FR propagator for simplicity. Here $\Phi(\mathbf{r}')$ and $\Psi(\bar{\boldsymbol{\rho}})$ are the exit field and the diffracted field, respectively, where $\mathbf{r}'$ denotes the intermediate plane coordinates and $\bar{\boldsymbol{\rho}}$ denotes the normalised (by a factor of λz) camera plane coordinates. Note that Eq. 29 is only valid under the paraxial approximation, i.e., $|\boldsymbol{\rho} - \mathbf{r}'| \ll \Delta z$. We can express $\Phi(\mathbf{r}')$ in the form of a 2D Fourier transform using the angular spectrum method:

$$\Phi(\mathbf{r}') = \iint \tilde{\Phi}(\mathbf{k}') e^{-i\mathbf{k}'\cdot\mathbf{r}'} \mathrm{d}^2\mathbf{k}' = \iint \tilde{\Phi}(\mathbf{k}') e^{-i\mathbf{K}'\cdot\mathbf{R}'|_{z'=0}} \mathrm{d}^2\mathbf{k}', \tag{30}$$

where $\mathbf{R}' = (\mathbf{r}', z)$ is the position vector and $\mathbf{K}' = (\mathbf{k}', k_z')$ is the corresponding wave vector with $\mathbf{k}' = 2\pi\bar{\boldsymbol{\rho}} = 2\pi\boldsymbol{\rho}/(\lambda z)$. Substituting the integral variable $\mathbf{k}'$ in Eq. 30 with $\mathbf{k}$, we can derive that

$$\Phi(\mathbf{r}') = \iint \tilde{\Phi}[\mathbf{k}'(\mathbf{k})] e^{i\mathbf{k}'(\mathbf{k})\cdot\mathbf{r}'} |\det \mathbf{J}_{\mathbf{k}'}(\mathbf{k})| \mathrm{d}^2\mathbf{k}, \tag{31}$$

where $\mathbf{k}'(\mathbf{k})$ is the non-linear 2D-mapping from $\mathbf{k} = (k_x, k_y)$ to $\mathbf{k}' = (k_x', k_y')$ that can be computed by using the 3D-transformation $\mathbf{K}' = \mathbf{TK}$, which can be derived with Eq. 25 and Eq. 28:

$$\mathbf{K}' \cdot \mathbf{R}' = (\mathbf{TK}) \cdot (\mathbf{TR}) = (\mathbf{TK}) \cdot \mathbf{R}', \tag{32}$$

and $|\det \mathbf{J}_{\mathbf{k}'}(\mathbf{k})|$ is the determinant of the Jacobian matrix, which is needed due to the integral variable substitution and is given by

$$|\det \mathbf{J}_{\mathbf{k}'}(\mathbf{k})| = \begin{vmatrix} \frac{\partial k_x'}{\partial k_x} & \frac{\partial k_x'}{\partial k_y} \\ \frac{\partial k_y'}{\partial k_x} & \frac{\partial k_y'}{\partial k_y} \end{vmatrix} = \frac{\partial k_x'}{\partial k_x}\frac{\partial k_y'}{\partial k_y} - \frac{\partial k_x'}{\partial k_y}\frac{\partial k_y'}{\partial k_x}. \tag{33}$$

Finally, by inserting Eq. 31 into Eq. 29, we obtain

$$\Psi(\bar{\boldsymbol{\rho}}) = \iint \left[\iint \tilde{\Phi}[\mathbf{k}'(\mathbf{k})] e^{i\mathbf{k}'(\mathbf{k})\cdot\mathbf{r}'} |\det \mathbf{J}_{\mathbf{k}'}(\mathbf{k})| \mathrm{d}^2\mathbf{k} \right] e^{-i2\pi\bar{\boldsymbol{\rho}}\cdot\mathbf{r}'} \mathrm{d}^2\mathbf{r}',$$
$$= \iint \tilde{\Phi}[\mathbf{k}'(\mathbf{k})] \left[\iint e^{-i2\pi\left[\bar{\boldsymbol{\rho}} - \frac{\mathbf{k}'(\mathbf{k})}{2\pi}\right]\cdot\mathbf{r}'} \mathrm{d}^2\mathbf{r}' \right] |\det \mathbf{J}_{\mathbf{k}'}(\mathbf{k})| \mathrm{d}^2\mathbf{k}. \quad (34)$$

As the integration of the exponential term leads to a Dirac delta function, we can further derive that

$$\Psi(\boldsymbol{\rho}) = \iint \tilde{\Phi}[\mathbf{k}'(\mathbf{k})] \delta \left[\frac{\boldsymbol{\rho}}{\lambda \Delta z} - \frac{\mathbf{k}'(\mathbf{k})}{2\pi} \right] |\det \mathbf{J}_{\mathbf{k}'}(\mathbf{k})| \mathrm{d}^2\mathbf{k}. \quad (35)$$

This formula indicates that the diffracted field Ψ equals the angular spectrum of the field in the intermediate plane $\tilde{\Phi}$. Consider a equally sampled grid $\mathbf{r}$ in the sample plane. We can generate the corresponding equidistantly sampled wave vector $\mathbf{k}$ and subsequently the non-equidistantly sampled wave vector $\mathbf{k}'$ with the following transformation $\mathbf{K}' = \mathbf{TK}$:

$$\begin{bmatrix} k'_x \\ k'_y \\ k'_z \end{bmatrix} = \begin{bmatrix} T_{11} & T_{12} & T_{13} \\ T_{21} & T_{22} & T_{23} \\ T_{31} & T_{32} & T_{33} \end{bmatrix} \begin{bmatrix} k_x \\ k_y \\ k_z \end{bmatrix}. \quad (36)$$

Consequently, we can express the mapping from $\mathbf{k} = (k_x, k_y)$ to $\mathbf{k}' = (k'_x, k'_y)$ as

$$k'_x(k_x, k_y) = T_{11}k_x + T_{12}k_y + T_{13}\sqrt{k_0^2 - k_x^2 - k_y^2}, \quad (37)$$

$$k'_y(k_x, k_y) = T_{21}k_x + T_{22}k_y + T_{23}\sqrt{k_0^2 - k_x^2 - k_y^2}, \quad (38)$$

where $k_z = \sqrt{k_0^2 - k_x^2 - k_y^2}$ with $k_0 = 2\pi/\lambda$ being the wave number. We can also derive that

$$\frac{\partial k'_x}{\partial k_x} = T_{11} - 2T_{13}\frac{k_x}{k_z}, \quad (39)$$

$$\frac{\partial k'_y}{\partial k_y} = T_{22} - 2T_{23}\frac{k_y}{k_z}, \quad (40)$$

$$\frac{\partial k'_x}{\partial k_y} = T_{12} - 2T_{13}\frac{k_y}{k_z}, \quad (41)$$

$$\frac{\partial k'_y}{\partial k_x} = T_{21} - 2T_{23}\frac{k_x}{k_z}, \quad (42)$$

which can be used for computing the determinant of the Jacobian $|\det \mathbf{J}_{\mathbf{k}'}(\mathbf{k})|$.

Note that $\tilde{\Phi}(\mathbf{k})$ and $\tilde{\Phi}(\mathbf{k}')$ are the diffraction patterns, which are presented by the same data matrix with different sampling grids. Namely, each point in the data matrix can be represented by locations in both sampling grids $\mathbf{k} = (k_x, k_y)$ and $\mathbf{k}' = (k'_x, k'_y)$. Meanwhile, Eq. 35 indicates that $\mathbf{k}'$ and $\boldsymbol{\rho}$ are both in the reciprocal space of the sample. Therefore, by interpolating from $\Psi(\boldsymbol{\rho})$ on a equidistant sampling grid to a $\Phi(\mathbf{k}')$ on a equidistant sampling grid, we can obtain the diffraction pattern $\tilde{\Phi}(\mathbf{k})$ in the plane parallel to the sample. This allows us to correct the diffraction pattern due to propagation between tilted planes.

7 Background subtraction and reconstruction

In the experiment, the background in all diffraction patterns originates from (1) the camera background noise and (2) the background signal due to spurious reflections in the beamline and specular reflection by the sample reaching the detector. Therefore, we measure the camera background noise in the absence of EUV illumination, which will be subtracted from each diffraction pattern prior to tilt correction, and retrieve the other background signal through ptychography reconstruction.

Fig. S6 shows that by subtracting the camera background noise, which is an almost uniform distribution, the details in the diffraction pattern (higher diffraction orders due to sample structures) can be revealed. The reconstructed background from all diffraction patterns can be interpreted as follows. Suppose that the field at the ellipsoidal mirror is given by

$$U(\mathbf{k}) = \mathrm{Pupil}(\mathbf{k}) U_{\mathrm{inc}}(\mathbf{k}), \tag{43}$$

where $\mathbf{k}$ represents the coordinate of the wave vector, conjugated with $\mathbf{r}$, the spatial coordinate in the sample plane. $U_{\mathrm{inc}}(\mathbf{k})$ is the incident field and $\mathrm{Pupil}(\mathbf{k})$ represents the pupil function of the ellipsoidal mirror. The probe that illuminates the sample can hence be written as

$$P(\mathbf{r}) = \mathcal{F}\left[U(\mathbf{k})\right](\mathbf{r}) \tag{44}$$

assuming that the sample is placed in the vicinity of the ellipsoidal mirror's focus and the focusing process can be interpreted by a Fourier transform. Consider a perfectly flat sample. The specular reflection of the probe by the sample that reaches the detector through free-space far-field propagation can be modelled by another Fourier transform:

$$U_{\mathrm{detector}}(\mathbf{k}) = \mathcal{F}\left[P(\mathbf{r})\right](\mathbf{k}). \tag{45}$$

Using the property of the double Fourier transform, we obtain that

$$U_{\mathrm{detector}}(\mathbf{k}) = \mathrm{Pupil}(-\mathbf{k}) U_{\mathrm{inc}}(-\mathbf{k}), \tag{46}$$

which implies that this signal conveys information about the focusing optics and the incident field. This signal can be interpreted as the signal reflected by

the flat substrate in reflection geometry. Because it remains constant during the scan, we can reconstruct it as a background from all diffraction patterns. In contrast, the signal reflected by the structures, varying per scanning position, contributes to the probe and object reconstruction. Fig. S7 shows a comparison between the diffraction pattern and the reconstructed background.

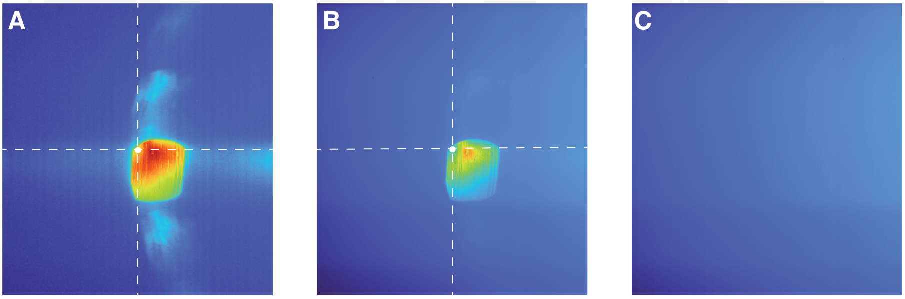

Fig. S6 Illustration of the raw diffraction pattern, averaged over 225 diffraction patterns used in the reconstruction and normalised to between zero and one, with (**A**) and without (**B**) background subtraction. The dashed lines indicate the horizontal and vertical axes and the solid dot represents the origin of the camera coordinate. The comparison shows that subtracting the camera background noise reveals the details of the diffraction pattern. (**C**): camera background noise.

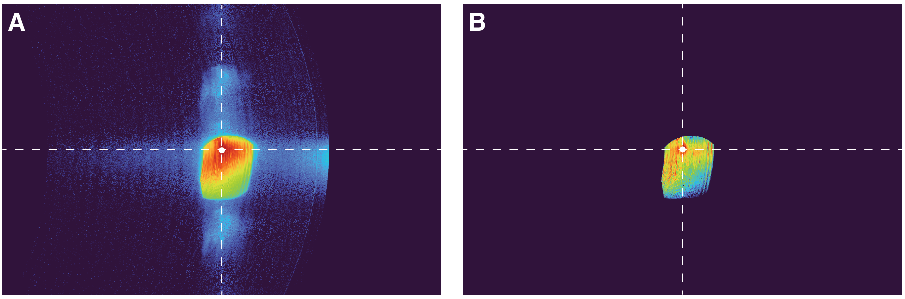

Fig. S7 Illustration of the diffraction pattern (**A**) and the reconstructed background (**B**). The dashed lines indicate the horizontal and vertical axes and the solid dot represents the origin of the camera coordinate. In the reconstruction, the diffraction pattern is corrected for tilted propagation, interpolated to enlarge the sample FOV, and thresholded to further suppress the noise. The background is reconstructed by updating the value at each pixel using a uniform distribution as the initial guess. The diffraction pattern and the background can be distinguished by the higher diffraction orders due to sample structures.

8 Optimiser and optimisation strategy

In optimisations, updating each variable along the direction of the gradient leads to the steepest descent of the loss function. Conventional gradient descent

optimiser updates the variable in a linear fashion by moving the variable along the direction of the gradient by a distance proportional to a step size such that

$$\boldsymbol{\theta}_i^{(j+1)} = \boldsymbol{\theta}_i^{(j)} + \alpha_i^{(j)} \frac{\partial \mathcal{L}}{\partial \boldsymbol{\theta}_i}(\boldsymbol{\theta}_i^{(j)}), \tag{47}$$

where $\alpha_i^{(j)}$ denotes the step size for the ith variable $\boldsymbol{\theta}_i$ in the jth iteration. We stop the update by specifying a number of iterations $j_{\max}$, which should guarantee at least one of the following conditions being reached:

1. the loss function is sufficiently minimised,
2. the gradient of the variable is sufficiently small such that the loss function cannot be minimised further.

To prevent the loss from oscillating in the vicinity of a minimum, we decay the step size exponentially at a constant rate:

$$\alpha_i^{(j)} = \beta_i^{\frac{j}{j_{\max}}} \alpha_i,\ j = 0, \cdots, j_{\max}, \tag{48}$$

where β_i is the constant decay rate and α_i is the initial step size for variable $\boldsymbol{\theta}_i$. Eq. 48 indicates that the step size decays exponentially from α_i to $\beta_i \alpha_i$.

Advanced momentum-based optimisers, e.g., the Adam optimiser [10], can improve the convergence of the loss function by preventing the rapid flipping of the update direction. The update scheme for these optimisers becomes

$$\boldsymbol{\theta}_i^{(j+1)} = \boldsymbol{\theta}_i^{(j)} + \alpha_i^{(j)} \Gamma \left[\frac{\partial \mathcal{L}}{\partial \boldsymbol{\theta}_i}(\boldsymbol{\theta}_i^{(0)}), \cdots, \frac{\partial \mathcal{L}}{\partial \boldsymbol{\theta}_i}(\boldsymbol{\theta}_i^{(j)}) \right], \tag{49}$$

where Γ represents the momentum operator for computing the update direction using gradients not only in the current iteration but also in the past iterations.

The variables have distinct physical interpretations and value scales, e.g., the probe and the object represent the field and the response of the sample to the field, respectively, while other variables often represent quantities with a dimension of lengths. As a result, each variable requires a unique step size and, optionally, a unique decay rate.

The momentum operator also applies to each variable individually. The choice of the length unit plays a significant role here. We chose the micrometre as our length unit instead of the SCI unit metre so that the length variables will not be either too small (wavelength) or too large (propagation distance) compared to the values of the probe and the object.

We use an empirical value $\alpha = 0.1$ as the default value of the step size for the probe and the object and $\beta = 0.99$ as the default value of the decay rate for all variables. These hyperparameters will be tuned during the optimisation by observing the curve of the loss function. We can also switch the update and the damping of each variable between on and off for optimised performance by tuning the value of α and β.

9 Regularisation and regularisation strategy

To convert ill-posed inverse problems into well-posed ones [11] and to improve the convergence, the optimisation needs to the regularised. In the developed algorithm, we introduce various regularisation terms to the loss function to impose our preference on the variables or to incorporate prior knowledge of variables into the optimisation. These regularisations often represent penalties or constraints on variables such as the probe and the object.

In computational imaging, we can choose among several regularisations to form a regularisation strategy, and we can express the resulting regularised loss function as:

$$\tilde{\mathcal{L}}(I_k, \hat{I}_k, \boldsymbol{\theta}_i) = \mathcal{L}(I_k, \hat{I}_k) + \sum\nolimits_h w_h \mathcal{R}_h(\boldsymbol{\theta}_i), \tag{50}$$

where w_h denotes the weight controlling the strength of the regularisation $\mathcal{R}_h$ on the variable $\boldsymbol{\theta}_i$. Eq. 50 is a sum of the data fidelity term $\mathcal{L}(I_k, \hat{I}_k)$, representing the original loss function, and several regularisation terms $\sum_h \gamma_h \mathcal{R}_h(\boldsymbol{\theta}_i)$. The gradients of the variables with respect to the regularised loss function can still be computed using the automatic-differentiation approach. We will discuss some choices of regularisations implemented in our ptychography algorithm here.

9.1 Regularisation using L1-norm

In the literature, the use of the L1-norm as a regularisation term was proposed by Terence Tao to recover information from incomplete and inaccurate measurements for compressive sensing [12]. L1-norm regularisation is particularly useful for sparse variables, which consist of only a few non-zero elements and mostly zeros elsewhere. Consider a 2-dimensional array of variable $\boldsymbol{\theta} = \{\theta_{p,q}\}$, where $\theta_{p,q}$ denotes its element with coordinate index (p, q). The L1-norm on $\boldsymbol{\theta}$ can be written as

$$\mathcal{R}_{L1}(\boldsymbol{\theta}) = w_{L1}\|\boldsymbol{\theta}\|_1 = w_{L1} \sum\nolimits_{p,q} |\theta_{p,q}|, \tag{51}$$

where w_{L1} is the weight of the regularisation. For complex-valued variables, we need to regularise separately the amplitude and the phase, both of which are real-valued functions.

Conventionally, the L1-norm regularised loss function is optimised by using a shrinkage-thresholding operator [13, 14] defined as

$$\tilde{\theta}_{p,q}^{L_1} = \begin{cases} \tilde{\theta}_{p,q} + w_{L_1}/2 & \text{if} \quad \tilde{\theta}_{p,q} \leq -w_{L_1}/2 \\ 0 & \text{if} \quad -w_{L_1}/2 < \tilde{\theta}_{p,q} < w_{L_1}/2 \\ \tilde{\theta}_{p,q} - w_{L_1}/2 & \text{if} \quad \tilde{\theta}_{p,q} \geq \ \ w_{L_1}/2 \end{cases}, \tag{52}$$

where $\tilde{\theta}_{p,q}$ is the variable updated by the gradient based on the loss function alone, and $\tilde{\theta}_{p,q}^{L_1}$ is the variable further updated by the shrinkage-thresholding operator for weight w_{L_1}. Eq. 52 indicates that the values of the variable will

either immediately be zero ($|\tilde{\theta}_{p,q}| < w_{L_1}/2$) or approaching zero at a constant speed of $w_{L_1}/2$ ($|\tilde{\theta}_{p,q}| \geq w_{L_1}/2$). For ptychography, the L1-norm regularisation is useful for enhancing the contrast between the signal and the background.

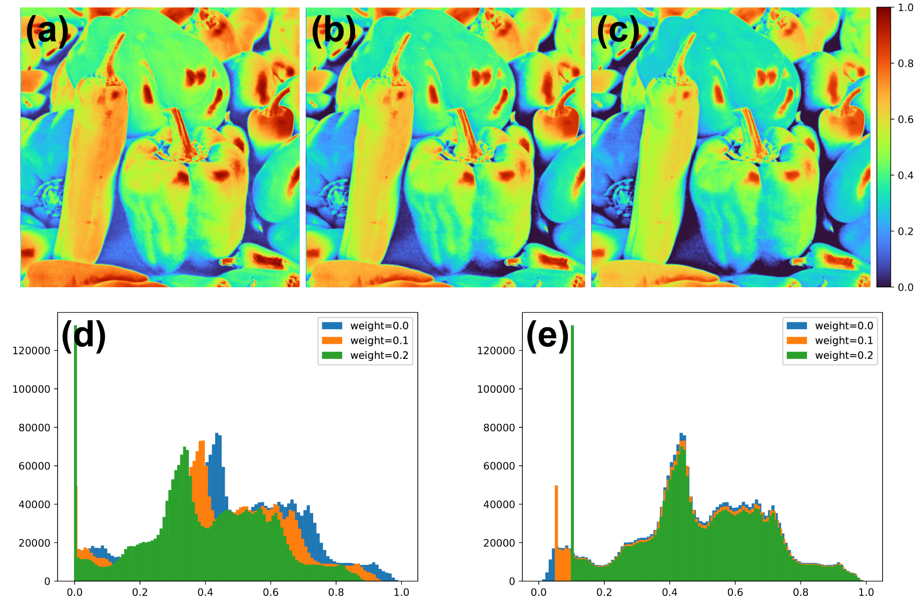

Fig. S8 Examples of using L1-norm regularisation for contrast enhancement with the image of "peppers" as the variable. Top: optimised variables for weight $w_{L1} = 0.0, 0.1, 0.2$. Bottom: histogram of the pixel value in the optimised variables for comparison. (e) is obtained by aligning the peaks in (d).

9.2 Regularisation using Total Variation (TV)

An intrinsic property of natural images is a certain amount of correlation between neighbouring pixels. This is usually not the case for noise. Therefore, total variation (TV), which is essentially a metric that applies the L1-norm to the spatial domain partial derivatives of the variable, has become one of the most popular regularisers in computational imaging. For a 2-dimensional discretised variable $\boldsymbol{\theta}(x, y) = \{\theta_{p,q}\}$, we define the anisotropic TV as

$$\begin{aligned} R_{TV}(\boldsymbol{\theta}) &= w_{TV} \left(\left\| \frac{\partial \boldsymbol{\theta}}{\partial x} \right\|_1 + \left\| \frac{\partial \boldsymbol{\theta}}{\partial y} \right\|_1 \right) \\ &= w_{TV} \sum\nolimits_{p,q} \left(|\theta_{p+1,q} - \theta_{p,q}| + |\theta_{p,q+1} - \theta_{p,q}| \right), \end{aligned} \tag{53}$$

where w_{TV} is the weight for the regularisation. Due to the use of the L1-norm, the TV regulariser suppresses noises in smooth areas with partial derivatives close to zero while maintaining the sharp boundaries with partial derivatives far from zero. The weight w_{TV} determines the threshold of the L1-norm

that applies to the partial derivatives of $\boldsymbol{\theta}$. TV regularisation is suitable for suppressing the noises in objects with piecewise constant structures. However, using TV regularisation may also force the structure of the object to be piecewise constant.

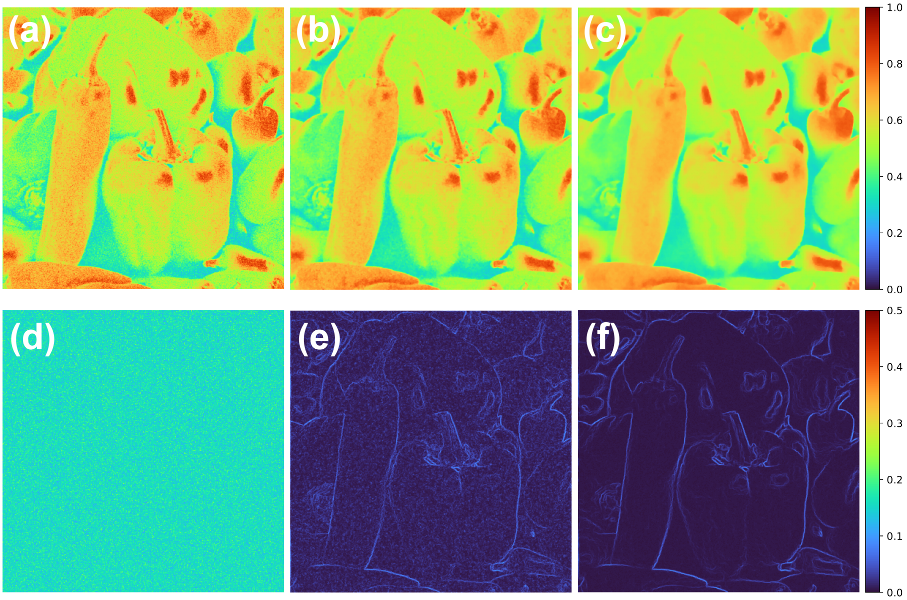

Fig. S9 Examples of using TV regularisation for noise suppression with the "peppers" image as the variable. Top: optimised variables for weight $w_{L1} = 0.0, 0.1, 0.2$. Bottom: histogram of the pixel value in the optimised variables for comparison.

9.3 Probe Support Constraint Regularization

Applying support constraints to the probe to avoid translation ambiguity is crucial in ptychography since the diffraction pattern $I(\boldsymbol{\rho})$ contains information of only the amplitude but not the phase. This means that if the exit field $\Phi(\mathbf{r})$ is a valid solution, an arbitrarily translated exit field $\Phi(\mathbf{r} - \Delta\mathbf{r})$ will also be a valid solution. $\Phi(\mathbf{r})$ and $\Phi(\mathbf{r} - \Delta\mathbf{r})$ yield the same diffraction pattern, but $\Phi(\mathbf{r} - \Delta\mathbf{r})$ yields an extra phase tilt compared to $\Phi(\mathbf{r})$ in the diffracted field. As a consequence, we can only uniquely determine the probe and the object up to a common translation.

To achieve convenient solutions for the probe and the object, we must confine the probe distribution in the area around the probe centre with a regularisation on the probe support, which is defined as

$$R_P(P) = w_P \sum\nolimits_{x,y} w_P(\mathbf{r}) |P(\mathbf{r})|^2, \tag{54}$$

where $w_P(\mathbf{r})$ is the spatial distribution of the weight defined as the product of a power function and a sigmoid function. The weighting function of the probe is given by

$$w_P(\mathbf{r}, \gamma_1, \gamma_2) = \frac{|\mathbf{r}|^{\gamma_1}}{1 + e^{-\gamma_2 |\mathbf{r}|}}, \tag{55}$$

where $|\mathbf{r}| = \sqrt{x^2 + y^2}$ denotes the radial coordinate, and γ_1 and γ_2 represent the power and the slope of the curve of the sigmoid function around the origin, respectively. We can incorporate the probe's centre (x_0, y_0), size (L_x, L_y), and rotation angle ϕ into the weighting function using the following transformation of the coordinates:

$$x' = \frac{(x - x_0)\cos(\phi) - (y - y_0)\sin(\phi)}{L_x}, \tag{56}$$

$$y' = \frac{(x - x_0)\sin(\phi) + (y - y_0)\cos(\phi)}{L_y}, \tag{57}$$

where $\mathbf{r} = (x, y)$ and $\mathbf{r}' = (x', y')$ are the coordinates before and after the transformation, respectively. The contour line of $|\mathbf{r}'| = 1$ represents an ellipse with center at (x_0, y_0), axes given by (l_x, l_y), and rotated by an angle of ϕ.

The sigmoid function equals to zero inside the contour line of $|\mathbf{r}'| = 1$ and one outside. The width of the transition area from zero to one is determined by the value of γ_2. In the meanwhile, the weighting function grows at a speed of $|\mathbf{r}'|$ to the power of γ_1 only in the area where the sigmoid function is non-zero. The weight distribution is determined solely by the sigmoid function when $\gamma_1 = 0$ and solely by the power function when $\gamma_2 = 0$.

10 Calculation of the deviation angle between harmonics

During the reconstruction, we guarantee that, for the two wavelengths case, both two probes are centred in the field-of-view with the probe support regularisation. However, when we align the two reconstructed objects at two wavelengths, we can observe shifted object field-of-views due to the relative shift between the two probes.

In order to quantitatively evaluate this relative shift, we compute the total intensity distribution in the scanned area on the sample, which is achieved by translating the intensity of the reconstructed probe to each scanning position and summing the total intensity of the translated probes. We further smooth the resulting intensity distribution with a Gaussian filter and we determine the location of the centre of mass with

$$x_{\mathrm{CM}} = \frac{\iint x A_{\mathrm{scan}}(x, y)\mathrm{d}x\mathrm{d}y}{\iint A_{\mathrm{scan}}(x, y)\mathrm{d}x\mathrm{d}y}, \tag{58}$$

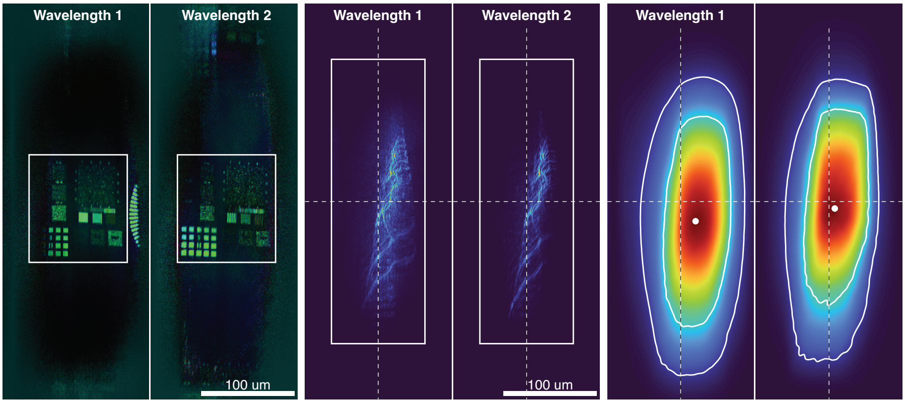

Fig. S10 From left to right: reconstructed objects, reconstructed probe intensities of all probe modes, and the intensity distribution in the scanned area on the sample, computed with the reconstructed probe intensities and the scanning positions. The white contours mark the areas illuminated by 75% and 95% EUV photons.

$$y_{\mathrm{CM}} = \frac{\iint y A_{\mathrm{scan}}(x, y)\mathrm{d}x\mathrm{d}y}{\iint A_{\mathrm{scan}}(x, y)\mathrm{d}x\mathrm{d}y}, \tag{59}$$

where $A_{\mathrm{scan}}(x, y)$ denotes the intensity distribution in the scanned area.

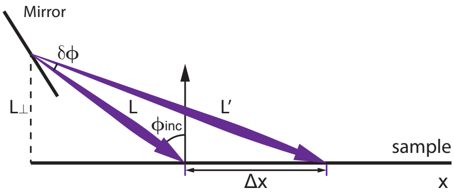

Fig. S11 Schematic illustration for the angular deviation between the propagation directions of the spectral harmonics. The propagation distances of the primary wavelength (used in the nominal design) and the other wavelength are denoted by L and L', respectively.

We find the relative shift between the centre of mass of the scanned area: $\Delta x_{\mathrm{CM}} = 13.60$ µm (horizontal) and $\Delta y_{\mathrm{CM}} = 9.85$ µm (vertical). Suppose that this relative shift is due to the deviation between the propagation directions of the illumination probes at the mirror, as shown in Fig. S11. For the primary wavelength at 17.93 nm, the nominal design of the illumination system considers a propagation distance of $L = 160$ mm from the mirror to the sample and an incidence angle at $\phi_{\mathrm{inc}} = 70°$ with respect to the sample surface normal. Assuming that the second wavelength at 17.30 nm propagates in the direction at an angle of $\delta\phi$ with respect to the primary wavelength, we can compute the propagation distance L' using the Law of Cosines:

$$L' = \sqrt{L^2 + \Delta x^2 - 2L\Delta x \cos\left(\phi_{\text{inc}} + \frac{\pi}{2}\right)} = 160.00127835 \text{ mm}, \tag{60}$$

and use the Law of Cosines again to compute the deviation angle:

$$\delta\phi_x = \arccos\left(\frac{L^2 + L'^2 - \Delta x^2}{2LL'}\right) = 0.0290775 \text{ mrad}. \tag{61}$$

11 Refocusing

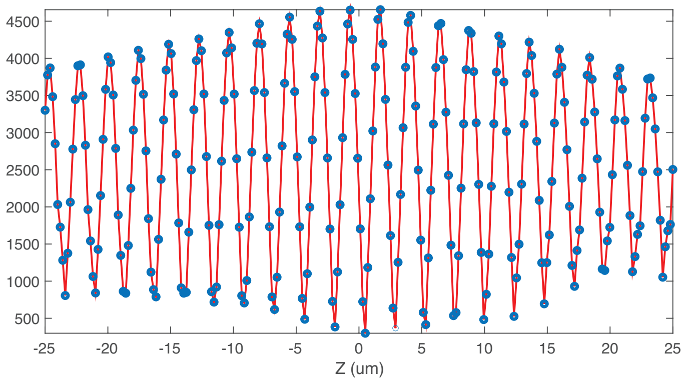

Fig. S12 Typical through-focus scan curve of gratings for finding the location of the focus. In this plot, the reconstructed object is propagated by a distance z using the angular spectrum method [7]. We crop the grating in the full field-of-view and plot the maximum value of its spatial frequency component between 4 µm^{-1} and 6 µm^{-1} versus the propagation distance z to obtain the oscillating curve with a varying envelop.

Because ptychography reconstructs complex-valued fields, we can propagate the reconstructed sample to find the focus of a particular area of interest and hence compensate the blurry due to errors in tilt correction (to certain extent) and sample curvature. To generate the plot illustrated in Fig. S12, we propagate the entire reconstructed sample by AS propagation described in Sec. 3.1 by a series of distances ranging from -25 µm to +25 µm. Then we crop the area of grating in the propagated sample and we find the maximum value of the spatial frequency component (obtained by computing the Fourier transform of the grating) between 4 µm^{-1} and 6 µm^{-1} for gratings with 200 nm pitch and 100 nm linewidth. The plot in Fig. S12 shows a fast oscillating curve (due to grating periodicity) with a slowly envelop (due to the non-periodic features, such as the line and gap pair in every pitch).

12 Orthogonal probe spatial modes

As discussed in Sec. 1, to consider the spatial partial coherence of the probe caused by HHG source instabilities, we need to use the 4-dimensional CSD instead of the 2-dimensional field. Notice that the CSD $J_P^\lambda(\mathbf{r}_1, \mathbf{r}_2)$ is actually a Hilbert-Schmidt kernel, whose eigendecomposition is given by

$$J_P(\lambda;\ \mathbf{r}_1, \mathbf{r}_2) = \sum_m P_m(\lambda;\ \mathbf{r}_1) P_m(\lambda;\ \mathbf{r}_2)^*, \tag{62}$$

where $P_m(\lambda;\ \mathbf{r})$ represents the mth probe spatial mode at wavelength λ and as a function of the sample plane coordinate $\mathbf{r}$. The span (the set of functions) $\{P_m(\lambda;\ \mathbf{r})\}$ forms a orthogonal basis (unnormalised) such that

$$\begin{aligned} \langle P_{m_1}(\lambda;\ \mathbf{r}), P_{m_2}(\lambda;\ \mathbf{r})\rangle &= \iint P_{m_1}(\lambda;\ \mathbf{r}) P_{m_2}(\lambda;\ \mathbf{r})^* \mathrm{d}\mathbf{r} \\ &= \begin{cases} |P_m|^2, & m_1 = m_2 \\ 0, & m_1 \neq m_2 \end{cases}, \end{aligned} \tag{63}$$

where <> denotes the inner product and $|P_m|^2$ denotes the total intensity of the mth spatial mode.

In practice, we assume only moderate spatial partial coherence. As a result, the CSD can be approximated by only a few spatial modes. Our algorithm requires a redefined number of spatial modes M to initialise a set of functions $\{P_m(\lambda;\ \mathbf{r})\}$. During the iterative optimisation, the original span $\{P_m(\lambda;\ \mathbf{r})\}$ will be replaced by another orthogonal span $\{\bar{P}_m(\lambda;\ \mathbf{r})\}$ obtained by using the method described in [15].

Consider matrices $\mathbf{P}$ and $\bar{\mathbf{P}}$, whose rows are 1-dimensional vectors obtained by stretching the 2-dimensional functions $P_m(\lambda;\ \mathbf{r})$ and $\bar{P}_m(\lambda;\ \mathbf{r})$, respectively. Both $\mathbf{P}$ and $\bar{\mathbf{P}}$ are matrices with size (M, N), where N is the total number of points on the sampling grid $\mathbf{r}$. The transformation from $\mathbf{P}$ to $\bar{\mathbf{P}}$ is given by

$$\bar{\mathbf{P}} = \mathbf{Q}\mathbf{P}, \tag{64}$$

where $\mathbf{Q}$ is a square matrix with size (M, M).

To find the transformation matrix $\mathbf{Q}$, we apply eigendecomposition to $\mathbf{P}\mathbf{P}^\dagger$, a square matrix with size (M, M), where $\dagger$ is the complex-conjugate transpose. The result can be written as

$$\mathbf{P}\mathbf{P}^\dagger = \mathbf{\Lambda} = \mathbf{Q}^\dagger \mathbf{\Sigma} \mathbf{Q}, \tag{65}$$

where the rows in $\mathbf{Q}$ are the eigenvectors of $\mathbf{\Lambda}$, and the diagonal elements of $\mathbf{\Sigma}$ are the corresponding eigenvalues. We can further derive that

$$\bar{\mathbf{P}}\bar{\mathbf{P}}^\dagger = (\mathbf{Q}\mathbf{P})(\mathbf{Q}\mathbf{P})^\dagger = \mathbf{Q}\mathbf{P}\mathbf{P}^\dagger\mathbf{Q}^\dagger = \mathbf{Q}\mathbf{Q}^\dagger\mathbf{\Sigma}\mathbf{Q}\mathbf{Q}^\dagger = \mathbf{\Sigma}. \tag{66}$$

Here we used the fact that the $\mathbf{Q}$ matrix is orthonormal ($\mathbf{Q}\mathbf{Q}^\dagger = \mathbf{I}$) to prove that the $\bar{\mathbf{P}}$ matrix is orthogonal ($\bar{\mathbf{P}}\bar{\mathbf{P}}^\dagger = \mathbf{\Sigma}$). The diagonal elements of $\mathbf{\Sigma}$ are the total intensities of the spatial modes $[|\bar{P}_1|^2, |\bar{P}_1|^2, \cdots, |\bar{P}_M|^2]$ as shown in

Eq. 63. The orthogonalisation process is sufficiently efficient in terms of both computation time and memory usage because the number of spatial modes M is small.

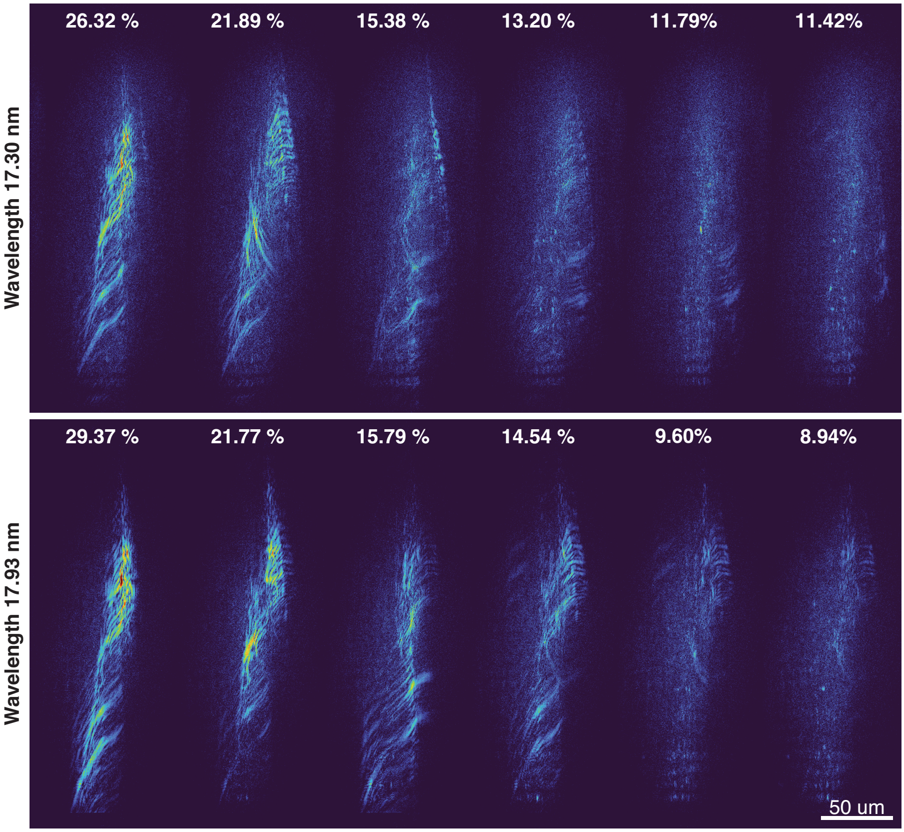

Fig. S13 Intensity distribution of the probe spatial modes. The percentage in each plot indicates the ratio between the energy of a particular spatial mode and the total probe. For each wavelength, the spatial modes are orthogonal with each other. This is guaranteed by orthogonalising the spatial modes every few iterations in the optimisation.

We plot the reconstructed probe spatial modes at two wavelengths in Fig. S13. We guarantee that the spatial modes at each wavelength are orthogonal with each other by using the described orthogonalising process every few iterations in the optimisation. The percentage number indicates the occupancy of a particular spatial mode, and the occupancy of the first mode can be interpreted as an indicator of the degree of spatial coherence. For coherent probes, typically the first mode dominates in the energy spectrum and the energy decays quickly from the first to the last mode.

For less coherent probes, the decay of mode energy is slower, meaning that we need more modes to properly approximate the CSD. We determine the

number of modes M by trial and error. We usually start with an initial guess of M based on our estimation of the probe coherence, and we find the smallest M that achieves a sufficiently small occupancy of the last mode by adjusting M through repeated reconstructions.

For the EUV probes shown in Fig. S13, the number of spatial modes M is restricted by the limited GPU memory. We can observe that these probes are significantly elongated in the sample tilt direction due to a 70° incidence angle. Furthermore, the first few spatial modes at each wavelength exhibit clear fine structures and speckles, while the last few spatial modes consist of only speckles.

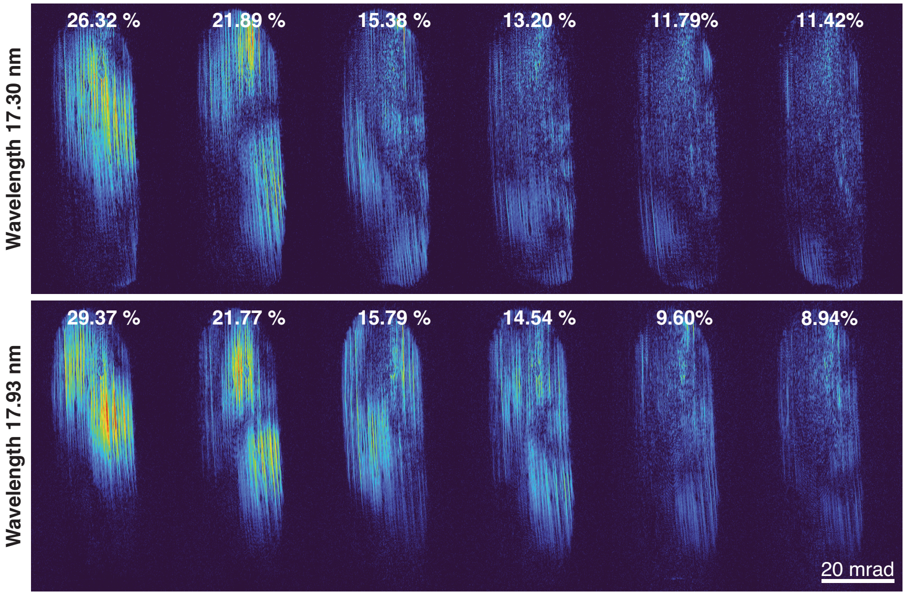

Fig. S14 Intensity distribution of the probe spatial modes in the pupil. The percentage in each plot indicates the ratio between the energy of a particular spatial mode and the total pupil field intensity.

We can obtain the EUV field in the pupil by applying the Fourier transform to each spatial mode. The structures and speckles in the probes are caused by the middle-frequency surface errors and the surface roughnesses, respectively, as shown in Fig. S14. It is also evident that spatial modes may represent the vibrational modes of the incident EUV beam because different spatial modes illuminate different pupil areas. In the reconstruction, we found that using multiple spatial modes improves the optimisation convergence and the quality of the reconstructed object and probe significantly.

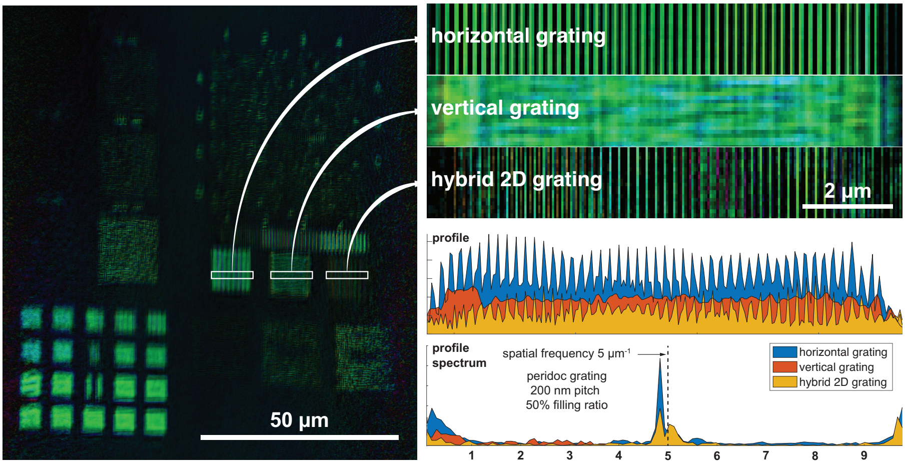

Fig. S15 Resolution determination by spatial frequency analysis. We obtain the profile in a region of interest in each grating and compute the profile spectrum by Fourier transformation. The location of the maximum spatial frequency component indicates the highest resolvable grating pitch.

13 Spatial frequency analysis for gratings

As illustrated in Fig. S15, we determine the resolution of the reconstructed sample by performing spatial frequency analysis on the gratings. We obtained the grating profile by summing the amplitude of the reconstructed grating in the 2D rectangle region of interest along the vertical direction. By Fourier transforming the profile along the horizontal direction, we computed the profile spectrum, which clearly shows that peaks located at spatial frequency 5 μm^{-1}, corresponding to a pitch of 200 nm and 50% filling ratio, for both horizontal grating and hybrid 2D grating. The vertical grating, however, shows a uniform profile and no peaks in the profile spectrum due to the asymmetric resolution of ptychography in reflection geometry.

14 Processing of reference AFM measurement

We used a Park NX20 atomic force microscope (AFM) to scan a specified region of interest on the sample (a part of the Siemens Star reconstructed through ptychography at 17.30 nm wavelength). The scanned area measures an area of 40-by-70 um with a spatial resolution of 156-by-273 nm. The AFM, in this particular setting, scans at a rate of 0.3 lines per second, leading to a total scan time of about 15 minutes.

We can separate the pixels representing the substrate and structures with a threshold. Although this separation is not accurate, this allows us to apply a 2-dimensional polynomial fit, 2 degrees in X and 3 degrees in Y directions, to the substrate data to accurately retrieve the background in the AFM image. By subtracting the background from the AFM image, we obtained the levelled AFM image as shown in Fig. S16(A).

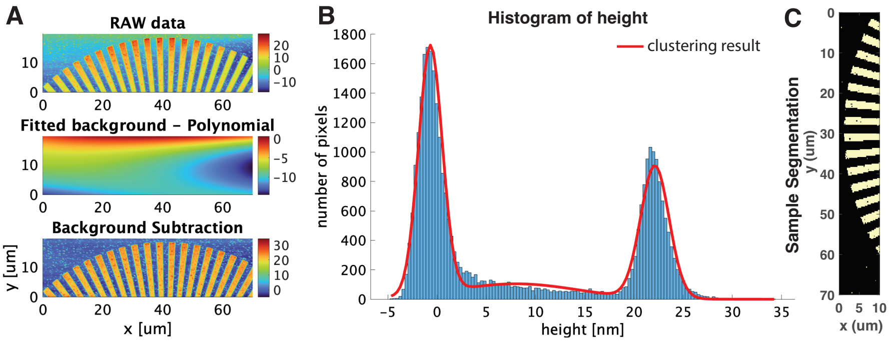

Fig. S16 Processing AFM measurement for structure height determination. **A**: Illustration of subtracting background, retrieved by polynomial fitting, from raw data to obtain levelled AFM image. **B**: Histogram for the number of pixels in the levelled AFM image as a function of height. **C**: Sample segmentation based on clustering the heights of the pixels.

To determine the height of the structures relative to the substrate, we apply Gaussian mixture model clustering (GMMc) to the levelled AFM image using the heights of pixels. Fig. S16(B) indicates that clustering finds the two peaks in the histogram (in Gaussian shapes), representing

- left peak: substrate with mean height -0.67 nm and variance 1.20 nm.
- right peak: structures with mean height 22.12 nm and variance 1.45 nm.

Therefore, the average relative height is 22.80 nm, which is in good agreement with the sample design. The segmented sample is plotted in Fig. S16(C).

15 Rigorous sample multilayer simulation

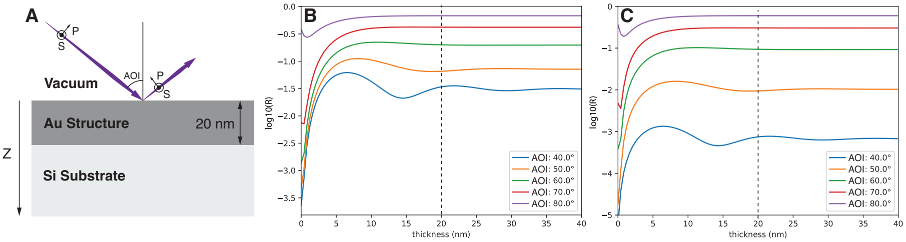

Fig. S17 Simulation multilayer layout of the sample (A), and the simulation results of reflectance in 10-base logarithm scale for the S (B) and P (C) polarisations versus a varying Gold layer thickness at various angles of incidence (AOI).

We study the behaviour of EUV light reflection on the sample through simulations using a rigorous multilayer model. This model is built based on the scattering matrix approach, in which the fields at the interfaces of layers are connected by the Redheffer star product. As a result, this model is numerically stable for simulating absorptive materials, evanescent wave propagation due

to total internal reflection, etc. We benchmarked the results of this model against the simulations on the CXRO (Center for X-ray Optics) database [16]. The refractive indices of Au and Si are $0.864 - 0.110i$ and $0.996 - 0.004i$, respectively, at 17.6 nm.

In the simulated multilayer shown by Fig. S17 (A), we assume semi-infinite spaces of vacuum at the top and Silicon at the bottom, and in between these two spaces, we placed a 20 nm layer of Gold. In Fig. S17 (B) and (C), we illustrate the simulation results for the S and P polarisations, respectively, for reflectance versus a varying thickness of the Gold layer at various angles of incidence (AOI). We can observe that at a 70-degree AOI, the reflectance increases monotonically as the thickness increases (red curve), and for 20 nm thickness, the reflectance has already been flat even in the logarithm scale plot, suggesting that the EUV light transmitted into the Gold layer through the vacuum-Gold interface has been absorbed completely and thus, never be reflected back to be able to interfere with the reflected EUV at the interface.

It is also shown in this figure that as we decrease the AOI, EUV light transmitted into the Gold layer increases. So, the transmitted EUV light can be reflected at the Gold-Silicon interface and reach again the Vacuum-Gold interface. This leads to several cycles of constructive and destructive interference as a function of the thickness until the Gold layer becomes so thick that all transmitted EUV light has been absorbed (orange and blue curves).

In summary, because there was no interference with the reflected EUV light occurring at the interface between the vacuum and the sample (valid for both Gold and Silicon) in our experiment, computing the reflection phase using the Fresnel coefficient can be justified. However, in other cases, the computation can be very complicated due to the single or even multiple internal reflections in the sample and a rigorous multilayer model is required. The scattering in the lateral plane is not considered in this work.

16 Relative height retrieval from reconstructed object phase

The relative height of the wafer structures can be retrieved from the phase of the reconstructed object. There are two main contributions to this phase:

- The reflection phase shift at the interface between vacuum and sample.
- The phase accumulated through light propagation.

Both phases depend on the angle of incidence (AOI) ϕ_{inc} and the wavelength λ. A schematic plot of the sample in reflection geometry is shown in Fig. S18. In the plane of reconstruction, the total phase difference between light reflected by the gold structures and the silicon substrate can be written as

$$\Phi_{\text{obj}}(\phi_{\text{inc}}, \lambda) = \Phi_{\text{Au}} - (\Phi_{\text{Si}} + \Phi_h) = \Delta\Phi - \Phi_h, \tag{67}$$

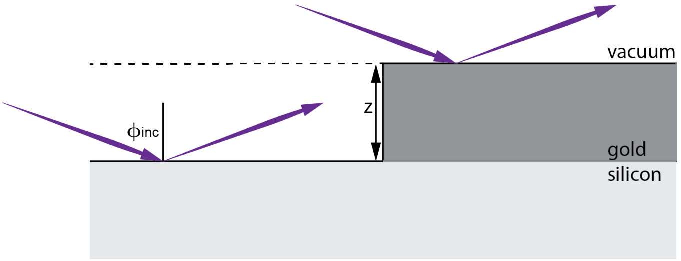

Fig. S18 Schematic plot of the sample used in our experiment. The illumination beam will be reflected by both the substrate (silicon) and the structures (gold). The difference between the sample phase is contributed by both the difference between the phase shifts, due to reflection at different interfaces, and the accumulative phases, due to light propagation over different distances.

where Φ_{Au} and Φ_{Si} denote the reflection phase shift at the Au and Si interfaces, respectively. For the computation, we used the refractive index data in [16] and interpolated it to obtain the refractive index at any wavelength. In our experimental design of the sample, the relative height of the Au structures on the Si substrate is 20 nm and the illumination beam is incident on the sample at 70° AOI.

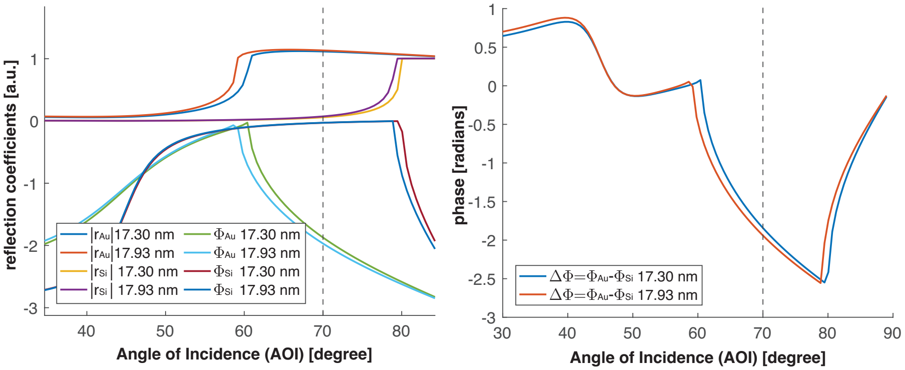

Fig. S19 Left: amplitude and phase of the reflection coefficients for plane waves as functions of incidence angle at the two considered wavelengths. Right: difference between the reflection phase shift at the Au and Si interfaces as functions of incidence angle.

We can compute the reflection phase shift at the interfaces as the phase of the complex-valued reflection coefficient using the Fresnel equations:

$$\begin{aligned} r_s &= \frac{n_i \cos\phi_{\mathrm{inc}} - n_t \cos\phi_{\mathrm{trn}}}{n_i \cos\phi_{\mathrm{inc}} + n_t \cos\phi_{\mathrm{trn}}} = |r_s| \exp\left(i\Phi_{r_s}\right), \\ r_p &= \frac{n_t \cos\phi_{\mathrm{inc}} - n_i \cos\phi_{\mathrm{trn}}}{n_t \cos\phi_{\mathrm{inc}} + n_i \cos\phi_{\mathrm{trn}}} = |r_p| \exp\left(i\Phi_{r_p}\right), \end{aligned} \tag{68}$$

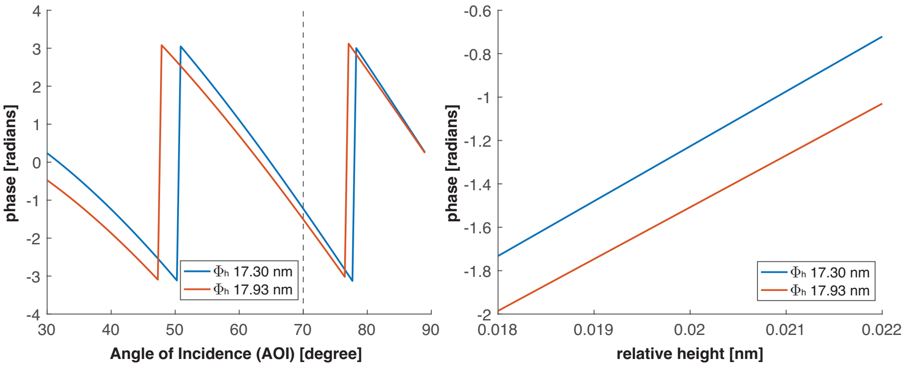

Fig. S20 Left: accumulative phase differences at the two considered wavelengths due to light propagation as functions of the incidence angle (wrapped to $[-\pi, +\pi]$) over a distance of height $h = 20$ nm along the z direction. Right: accumulative phase differences for 70° AOI as functions of the height h.

where r_s and r_p are the reflection coefficients for the s and p polarisations, respectively. n_i and n_t are the refractive indices of the vacuum and the sample, and ϕ_{trn} is the angle of transmission given by

$$\phi_{\text{trn}} = \left| \arcsin\left(\frac{n_i \sin\phi_{\text{inc}}}{\Re\{n_t\}}\right)\right|. \tag{69}$$

Notice that only the real part of the refractive index is used when the medium is lossy, and the absolute value guarantees that Eq. 69 is always valid even when total internal reflection occurs. In Eq. 68, because the medium is dispersive, the reflection phase shift depends on the wavelength λ. Since the variation of the reflection coefficient is smooth as a function of the incidence angle (as indicated by Fig. S19) and the numerical aperture of the illumination system is small (about 40 mrad), we only need to consider the reflection in response to a plane wave with nominal AOI at $\phi_{\text{inc}} = 70°$.

Considering light propagating at ϕ_{inc} with respect to the surface normal of the sample, the difference between the accumulative phase due to the reflection at the Au and Si interface can be written as

$$\Phi_h = 2k_z h = 2\frac{2\pi}{\lambda}\sin\left(\frac{\pi}{2} - \phi_{\text{inc}}\right)h, \tag{70}$$

where the factor 2 accounts for two-pass the upward and the downward propagation, k_z is the z component of the wave vector $\|k\| = 2\pi/\lambda$, and h is the relative height. Once we compute the phase shift difference $\Delta\Phi$ with Eq. 68, we can obtain the accumulative phase difference as $\Phi_h = \Phi_{\text{obj}} - \Delta\Phi$. However, Fig. S20 shows that the height cannot be determined uniquely due to phase wrapping. As a result, we need the design value of the height to determine the actual height value in the vicinity or need two or more wavelengths to resolve the ambiguity. Besides, careful calibration of AOI is crucial, which determines the reflection phase shift difference $\Delta\Phi$.

17 Error analysis against mixed noise and systematic error

In this section, we investigate the accuracy and precision of phase retrieval against mixed Gaussian and Poisson noise and system errors, such as the error of propagation distance. To generate the simulation dataset, we convert the SEM image to a binary object. The gold structures and the silicon substrate have 1.0000 and 0.0820 amplitudes and 1.6494 and -0.0001 phases, respectively, based on the reflection coefficients computed for 18 nm EUV illumination and 20 degrees of grazing incidence angle. In the simulation, we consider a single wavelength and spatial mode reconstructed from the experimental dataset.

To simulate the mixed noise, we normalised the entire scanning series by the maximum total energy of the diffraction patterns. In Fig. S21 (A), we show the diffraction pattern whose total energy, which previously was the maximum in the scanning series, was normalised to unity. The maximum total energy in our experimental dataset (dark background subtracted) is 2×10^9 in terms of photons, which is about only 10% of the photons measured at the source (2×10^{11} photons per second) for 100 ms exposure time.

To simulate the effect of Poisson noise, we scale the maximum total energy in the scanning series to 5×10^6 and 5×10^7 photons, equivalent to a fraction of $\frac{1}{4000}$ and $\frac{1}{400}$ source energy, respectively. We then add Gaussian noise with a mean of 400 and a standard derivation of 15 photons, as shown in Fig. S21 (B), to the simulated diffraction patterns. This Gaussian noise statistic is determined based on our measurement of the camera background signal. The resulting diffraction patterns with mixed Gaussian and Poisson noise are shown in Fig. S21 (C) and (D) for a total number of 5×10^6 and 5×10^6 photons, respectively.

In the reconstruction, we subtract the Gaussian noise mean (400 photons) from the diffraction patterns. As a result, some pixel values will inevitably become negative. For loss functions using the modulus of the diffracted field, these pixel values need to be clipped at a non-negative value. In this analysis, we performed eight reconstructions with the same optimisation settings on different noise realisations for each case.

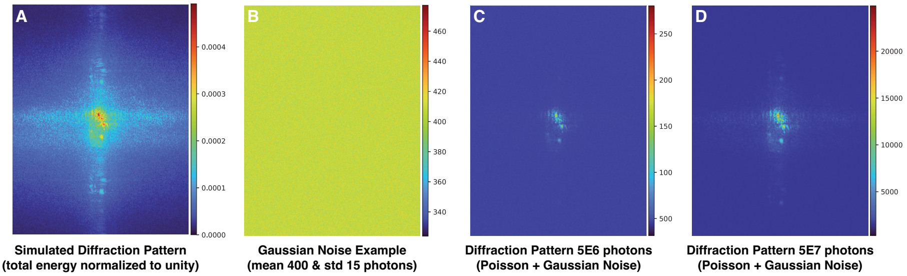

Fig. S21 Illustration of simulated datasets. (A) Simulated noise-free diffraction pattern. (B) Gaussian noise with a mean of 400 and a standard derivation of 15 photons. (C) and (D): Simulated noisy diffraction pattern with a photon number of 5×10^6 and 5×10^7.

Prior to analyzing the reconstructed amplitude and phase, it is important to preprocess the results. Due to the shift ambiguity of the probe and object, we need to align different reconstructed objects, based on the cross-correlation, to select the same region-of-interest (ROI). Fig. S22 shows the full object (A) before and the selected ROI (B) after the alignment procedure. We also need to correct other artefacts such as the tilt, which is an effect of the shift ambiguity, and the offset in the reconstructed phase. The tilt and offset correction for the reconstructed phase is illustrated in Fig. S23. We can see that the final results will now be ready for analysis.

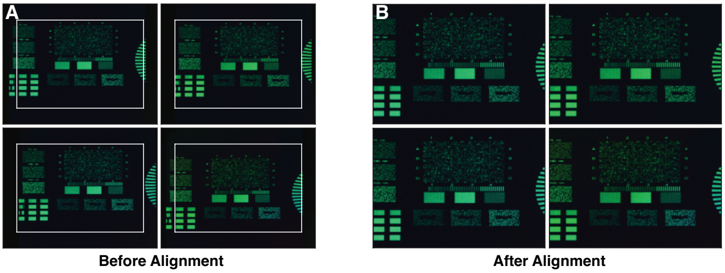

Fig. S22 Reconstructed object in complex plot before (A) and after (B) the alignment using cross-correlation.

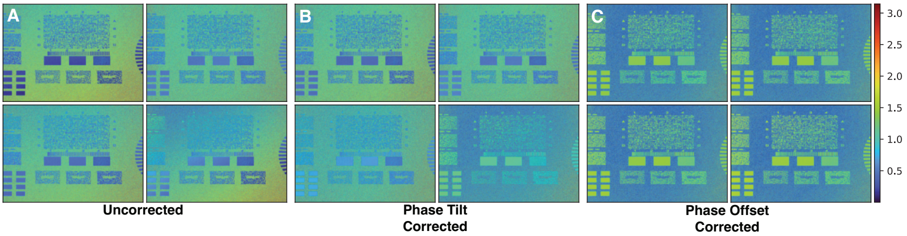

Fig. S23 Original uncorrected object phase (A) and the results of tilt (B) and offset (C) correction. This correction is important for achieving consistent results.

Following the approach in this article, we select the ROI of the Siemens star to determine the relative hight of gold structure relative to silicon substrate. Although the reconstructed object does not show any noticeable differences in the complex plot as illustrated in Fig. S24 (A), we do observe the difference in the phase due to illumination using different numbers of photons in Fig. S24 (B).

We can further observe in the amplitude-phase histogram plot Fig. S24 (D) that by increasing the photon number in the illumination while maintaining the noise level, we can achieve a increased contrast and decreased variation in the amplitude and phase, respectively. When applying Gaussian Mixture Model (GMM) segmentation to the reconstructed object using three clusters,

we can identify the silicon substrate (purple), the gold structure (yellow), and a transition region (green) separated in distinct clusters. The transition region can be found in Fig. S24 (C) at the edges of the Siemens Star.

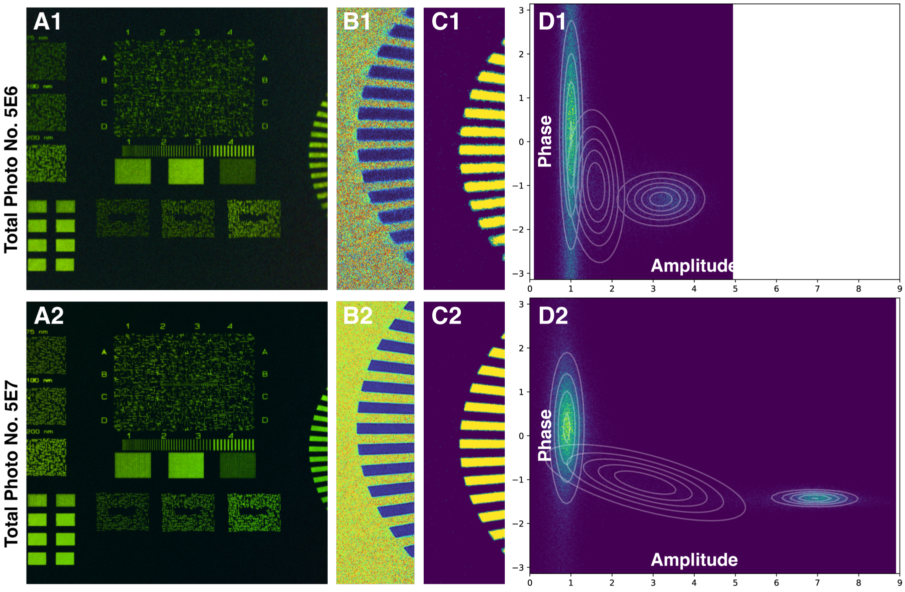

Fig. S24 Comparison of Gaussian Model Mixture (GMM) segmentation results for two cases of 5×10^6 (top) and 5×10^7 (bottom) photons. (A) Reconstructed sample in complex plot. (B) The ROI of Siemens star in phase plot. (C) Siemens star segmented by applying GMM to the amplitude-phase histogram (purple: Si substrate, yellow: Au structure, and green: transition region). (D) The amplitude-phase histogram of Siemens star. The contour lines show the Gaussian distributions of the three clusters

The mean and standard derivation of the Gaussian distributions in GMM segmentation offers insights into the accuracy and precision. We obtained an average phase difference of 1.649 and 1.617 and combined standard derivation (mostly contributed by the substrate) of 0.850 and 1.450 for 5×10^6 and 5×10^7 number of photons, respectively. These results indicate that noise does NOT impact the accuracy of the relative phase but does impact the precision. As we can observe in Fig. S24 (D), compared with Au structure, Si substrate shows higher phase variance due to lower amplitude values. Meanwhile, we observe that as the photon number in the illumination decreases, the phase spread of both clusters increases significantly. These results confirm our observation in the experimental dataset that the main challenge for phase determination is to find a reference. In EUV metrology, this reference should reflect sufficient EUV light, and its material and height must be carefully calibrated.

To analyse the robustness of phase determination against both mixed noise and system error, we deviate the propagation distance in our AD model by

Table 1 Phase Difference Determination Results for 5×10^6 photons

	1	2	3	4	5	6	7	8	mean	std
mean phase difference	1.964	1.528	1.616	1.643	1.938	2.044	1.230	1.230	1.649	3.18E-01
std substrate phase	1.131	1.386	1.329	1.373	1.202	1.186	2.464	1.867	1.492	4.55E-01
std structure phase	0.140	0.171	0.192	0.176	0.153	0.134	0.117	0.117	0.150	2.76E-02

Table 2 Phase Difference Determination Results for 5×10^7 photons

	1	2	3	4	5	6	7	8	mean	std
mean phase difference	1.598	1.627	1.600	1.602	1.581	1.664	1.671	1.592	1.617	3.37E-02
std substrate phase	0.899	0.777	0.888	0.807	0.967	0.741	0.966	0.757	0.850	9.16E-02
std structure phase	0.012	0.014	0.012	0.013	0.013	0.014	0.012	0.012	0.013	8.65E-04

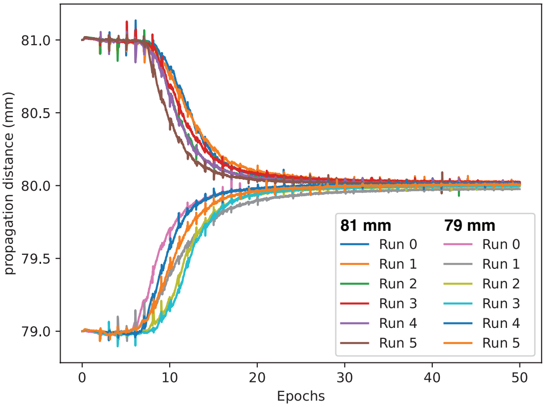

Fig. S25 Progress of propagation distance correction during the reconstruction. Each epoch consists of 202 batches with a unit batch size, and we optimised for 50 epochs in each run.

±1 mm from the ground truth. We repeat six reconstructions with the same optimisation settings on different noise realisations based on 5×10^7 photons for deviations in both directions. Note that changing the propagation distance also leads to a corresponding change in the sampling of the probe and object. Fig. S25 shows that the propagation distance is corrected alongside the reconstruction of the probe and object, as indicated by the smoothly varying curve, through a unified update scheme allowed by AD. All reconstructions, starting from both above (81 mm) and below (79 mm), converge to the ground truth of 80 mm within 50 iterations. In Tab. 3, we have achieved the final correction results of 80.020 mm ±6 µm from 81 mm and 79.998 mm ±13 µm from 79 mm. This verifies that millimetre-level propagation distance errors can be corrected through optimization by our algorithm.

Comparing the results in Tab. 4 (81 mm) and Tab. 5 (79 mm) with the original phase determination results in Tab. 2, we found that by correcting the propagation distance error, the mean of the relative phase has restored to that of the undisturbed case. The slightly larger standard derivation might be due to the sampling change, which causes the sample no longer to be binary.

Table 3 Propagation Distance Correction Results

initial value	1	2	3	4	5	6	mean	std
81 mm	80.017	80.025	80.026	80.026	80.017	80.011	80.020	0.006
79 mm	80.008	79.976	79.992	79.999	80.010	80.002	79.998	0.013

Table 4 Phase Determination Results for 81 mm Propagation Distance

initial value	1	2	3	4	5	6	mean	std
mean phase difference	1.620	1.626	1.623	1.616	1.610	1.612	1.618	6.30E-03
std substrate phase	1.010	1.055	1.021	1.081	1.061	1.022	1.042	2.78E-02
std structure phase	0.011	0.012	0.011	0.012	0.011	0.010	0.011	6.45E-04

Table 5 Phase Determination Results for 79 mm Propagation Distance

initial value	1	2	3	4	5	6	mean	std
1.623	1.597	1.596	1.615	1.655	1.611	1.616	2.19E-02	6.30E-03
1.113	1.157	1.088	1.101	1.088	1.136	1.114	2.79E-02	2.78E-02
0.013	0.013	0.012	0.012	0.011	0.012	0.012	7.43E-04	6.45E-04

18 Determining the height of wafer structures

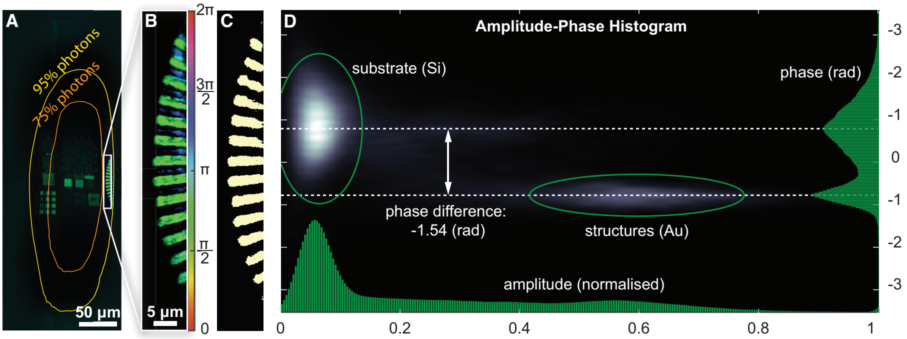

Fig. S26 Left: the reconstructed complex-valued object at wavelength $\lambda = 17.30$ nm and zoomed view of the Siemens star. Right: amplitude-phase histogram of the pixels that the Siemens star consists of.

In Fig. S26, the Siemens star exhibits two distinct pixel clusters in the histogram plotted in amplitude-phase coordinates. The amplitude and phase histograms are also shown in the figure, attached to the respective axes. We use the GMMc method to separate the two clusters, using as a distance metric the amplitude phase values and the location of each pixel in the reconstructed object. Compared with the k-means clustering method, which draws a straight line between the clusters, the GMMc method decides which cluster a pixel belongs to based on the probability and fits both the mean and variance values and the orientation of the Gaussian probability distributions, depicted by the green circles in Fig. S26.

For the case shown in Fig. S26, the difference between the average phase is $\Phi_{\text{obj}} = -1.54$ rad. Subtracting the reflection phase shift difference at the interface $\Delta\Phi = -1.92$ rad, we obtain the propagation accumulative phase difference given by $\Phi_h = \Delta\Phi - \Phi_{\text{obj}} + 2\pi = 5.90$ rad, and we can determine retrieve the height using

$$z = \frac{\lambda}{2\pi} \frac{\Phi_h}{2\sin\left(\frac{\pi}{2} - \phi_{\text{inc}}\right)} = 23.60 \text{ nm}. \tag{71}$$

with wavelength $\lambda = 17.3$ nm and incident angle $\phi_{\text{inc}} = 70°$. The variance of the structure phase is 0.15 rad, corresponding to a height variance of $\Delta z = 0.62$ nm. Although the substrate cluster is still sharply peaked in both the amplitude and phase histograms, the height variance is relatively large ($\Delta z = 2.65$ nm) due to the low intensity. Nevertheless, the retrieved height agrees well with both the sample design and the AFM measurement.

References

[1] Wolf, E.: New theory of partial coherence in the space–frequency domain. part i: spectra and cross spectra of steady-state sources. JOSA **72**(3), 343–351 (1982)

[2] Rabiner, L., Schafer, R.W., Rader, C.: The chirp z-transform algorithm. IEEE transactions on audio and electroacoustics **17**(2), 86–92 (1969)

[3] Rabiner, L.R., Gold, B.: Theory and application of digital signal processing. Englewood Cliffs: Prentice-Hall (1975)

[4] Hu, Y., Wang, Z., Wang, X., Ji, S., Zhang, C., Li, J., Zhu, W., Wu, D., Chu, J.: Efficient full-path optical calculation of scalar and vector diffraction using the bluestein method. Light: Science & Applications **9**(1), 1–11 (2020)

[5] Shao, Y., Urbach, H.P.: Comment on "efficient full-path optical calculation of scalar and vector diffraction using the bluestein method". Light: Science & Applications **10**(1), 1–1 (2021)

[6] Shen, F., Wang, A.: Fast-fourier-transform based numerical integration method for the rayleigh-sommerfeld diffraction formula. Applied optics **45**(6), 1102–1110 (2006)

[7] Matsushima, K., Shimobaba, T.: Band-limited angular spectrum method for numerical simulation of free-space propagation in far and near fields. Optics express **17**(22), 19662–19673 (2009)

[8] Matsushima, K., Schimmel, H., Wyrowski, F.: Fast calculation method for optical diffraction on tilted planes by use of the angular spectrum of plane waves. JOSA A **20**(9), 1755–1762 (2003)

[9] Matsushima, K.: Introduction to Computer Holography: Creating Computer-Generated Holograms as the Ultimate 3D Image. Springer, ??? (2020)

[10] Kingma, D.P., Ba, J.: Adam: A method for stochastic optimization. arXiv preprint arXiv:1412.6980 (2014)

[11] Goodfellow, I., Bengio, Y., Courville, A.: Deep Learning. MIT press, ??? (2016)

[12] Candes, E.J., Romberg, J.K., Tao, T.: Stable signal recovery from incomplete and inaccurate measurements. Communications on Pure and Applied Mathematics: A Journal Issued by the Courant Institute of Mathematical Sciences **59**(8), 1207–1223 (2006)

[13] Daubechies, I., Defrise, M., De Mol, C.: An iterative thresholding algorithm for linear inverse problems with a sparsity constraint. Communications on Pure and Applied Mathematics: A Journal Issued by the Courant Institute of Mathematical Sciences **57**(11), 1413–1457 (2004)

[14] Beck, A., Teboulle, M.: A fast iterative shrinkage-thresholding algorithm for linear inverse problems. SIAM journal on imaging sciences **2**(1), 183–202 (2009)

[15] Li, P., Edo, T., Batey, D., Rodenburg, J., Maiden, A.: Breaking ambiguities in mixed state ptychography. Optics express **24**(8), 9038–9052 (2016)

[16] Henke, B.L., Gullikson, E.M., Davis, J.C.: X-ray interactions: photoabsorption, scattering, transmission, and reflection at e= 50-30,000 ev, z= 1-92. Atomic data and nuclear data tables **54**(2), 181–342 (1993)
